# Large High‐Temperature Piezoelectric Response of Lead‐free BiFeO_3_–BaTiO_3_ Originating from Relaxor Disorder

**DOI:** 10.1002/smll.202502379

**Published:** 2025-06-09

**Authors:** Antonio Iacomini, Janina Roknić, Issa Sidibe, Marco Scavini, Mojca Otoničar, Hana Uršič, Tadej Rojac

**Affiliations:** ^1^ Electronic Ceramics Department Jožef Stefan Institute Ljubljana 1000 Slovenia; ^2^ Jožef Stefan International Postgraduate School Ljubljana 1000 Slovenia; ^3^ FST department University of Lille EEA 59650 Villeneuve d'Ascq Lille 59650 France; ^4^ Department of Chemistry University of Milan Via Golgi 19 Milano 20133 Italy

**Keywords:** BFO‐BTO, domains, nonlinearity, piezoelectric, relaxor

## Abstract

To date, no piezoelectric material system has been developed that would match the piezoelectric performance of the group of lead‐based relaxor ferroelectrics, such as Pb(Mg_1/3_Nb_2/3_)O_3_–PbTiO_3_ (PMN–PT). In the quest to find alternatives, continuous efforts have been made to understand the complex microscopic origins leading to the large macroscopic piezoelectric response of PMN–PT and similar lead‐based perovskites with relaxor characteristics. Despite significant advances, it is still unclear whether those concepts can be efficiently used to develop lead‐free relaxor ferroelectric alternatives. Here, a large piezoelectric response of BiFeO_3_–BaTiO_3_ (BFO–BTO) ceramics is reported, characterized by a d_33_ coefficient of 1200 pm V^−1^ measured at 325 °C, 1 kV cm^−1^ of driving field and 90 Hz of field frequency. While composed of multiple contributions, the large response is found to be dominated by a strongly nonlinear and hysteretic process, likely associated with the dynamics of hierarchically arranged nanodomain walls, thus resembling the mechanisms operating in lead‐based relaxor materials. Although the response is triggered upon elevated thermal energy, the results provide valuable information about the microscopic behavior that can be potentially used to tailor the response of lead‐free relaxor ferroelectrics and bring them closer to the highly performant lead‐based perovskites.

## Graphical abstract

In situ, temperature‐dependent analysis of the piezoelectric response of the 0.67BFO‐0.33BTO system reveals an exceptionally high piezoelectric response of ≈1200 pm V^−1^. Detailed temperature‐dependent examination uncovers three distinct stages, mutually contributing to the overall response. This study provides valuable insights into the piezoelectric behavior of the material across different temperature ranges, enhancing the understanding of its potential applications.

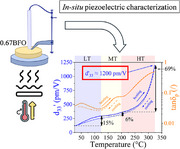

## 1 Introduction

Relaxor ferroelectric oxide perovskites, exemplified by Pb(Mg_1/3_Nb_2/3_)O_3_–PbTiO_3_ (PMN–PT), have been the focus of continuous scientific investigation for over six decades. These materials have garnered significant attention due to their extraordinary piezoelectric properties, with, e.g., longitudinal piezoelectric d_33_ coefficients in ceramics, reaching levels up to ≈700 pC/N close to the so‐called morphotropic phase boundary (MPB) ^[^ ^1^, ^2^ ^]^ or even ≈1500 pC/N when doped with samarium ions.^[^ ^3^ ^]^ Such remarkable performance surpasses that of the commercially prevalent Pb(Zr,Ti)O_3_ (PZT).^[^ ^3^, ^4^ ^]^ Despite the extensive research on these complex materials, it is still not completely understood why PMN–PT exhibits superior properties compared to PZT. The consensus based on diverse independent studies is that the difference comes from the relaxor nature of PMN–PT.^[^ ^5^, ^6^, ^7^, ^8^, ^9^, ^10^ ^]^ While most of those studies have been focused on PMN–PT compositions close to MPB, recently, Otoničar et al.^[^ ^10^ ^]^ have proposed that the highly mobile low‐angle nanodomain walls, arranged hierarchically inside microdomains, are the critical domain‐structure elements responsible for the exceptionally large nonlinear and hysteretic piezoelectric and dielectric responses observed in monoclinic PMN–PT compositions away from the MPB. On the other hand, it appears that a single mechanism cannot plausibly explain all the details of the large piezoelectric response of these complex materials. For example, a recent study by Arzenšek et al.^[^ ^11^ ^]^ has demonstrated that the origin of the piezoelectric response in Sm‐doped PMN–PT is actually the result of multiple and emergent microscopic mechanisms, including coupled lattice (intrinsic) and non‐lattice (extrinsic) effects associated with the relaxor disorder. To date, it is still not clear whether the mechanisms of piezoelectric enhancement related to the relaxor nature of PMN–PT can be efficiently used to engineer and enhance properties of lead‐free relaxor ferroelectrics.

Among lead‐free piezoelectric ceramic systems, the BiFeO_3_–BaTiO_3_ (BFO–BTO) solid solution has emerged as the most promising relaxor ferroelectric material, garnering significant attention for high‐temperature applications due to its elevated Curie temperature (*T_c_*), which typically exceeds 400 °C (*T_c_* depends on the exact composition).^[^ ^12^, ^13^ ^]^ Both unmodified BFO and BTO are ferroelectric, however, a tendency to form a relaxor‐like phase was observed by progressively adding BTO when forming a solid solution with BFO.^[^ ^13^, ^14^, ^15^, ^16^, ^17^, ^18^ ^]^ Based on previous reports, the most interesting composition in terms of the piezoelectric response contains ≈33 mol% of BTO, corresponding to the region in the phase diagram where the transition from a rhombohedral to a pseudocubic structure is commonly observed.^[^ ^12^, ^19^, ^20^ ^]^ Nonetheless, the room‐temperature piezoelectric properties are modest, on the level of 100 pC/N,^[^ ^12^, ^13^, ^21^, ^22^, ^23^, ^24^, ^25^ ^]^ thus significantly lower than those typical for lead‐based relaxor ferroelectric systems, such as PMN–PT. The intriguing aspect of BFO–BTO is that its piezoelectric coefficient increases with temperature. Independent studies have reported that BFO–BTO‐based materials exhibit remarkably high piezoelectric coefficients, ranging between 250 and 750 pC/N, in the temperature range of 250–450 °C.^[^ ^17^, ^26^, ^27^, ^28^, ^29^, ^30^, ^31^ ^]^ It has to be emphasized that the majority of those studies report on very complex compositions where BFO–BTO (with limited variations in the BTO content) is mixed with several other perovskite solid solutions, such as Bi(Zn_0.5_Ti_0.5_)O_3_,^[^ ^27^ ^]^ Bi(Ni_2/3_Nb_1/3_)O_3_,^[^ ^28^ ^]^ Bi(Zn_0.5_Hf_0.5_)O_3_,^[^ ^17^ ^]^ (Bi_0.5_Na_0.5_)TiO_3_^[^ ^31^ ^]^ or even pseudo‐binary 0.8(Bi_0.5_Na_0.5_)TiO_3_–0.2(Bi_0.5_K_0.5_)TiO_3_.^[^ ^26^ ^]^ Clearly, complicated chemical compositions with multiple cations necessarily make the interpretations of the large coefficients difficult, if not impossible. Nevertheless, despite proper experimental support for the interpretation, the significant high‐temperature coefficient was pragmatically explained by the presence of a ferroelectric state exhibiting nanoscale structural heterogeneity within the so‐called “composite” domain structure, where nanodomains and polar nanoregions (PNRs) coexist.^[^ ^26^, ^27^ ^]^ On the other hand, Xie et al.^[^ ^29^ ^]^ succeeded in separating the linear and nonlinear contributions to the direct piezoelectric response of BFO–BTO. Based on their systematic analysis they provided a possible explanation of the temperature‐dependent piezoelectric response revolving around the relaxor features of the system. Even in this case, however, several other possibilities were underestimated or neglected, such as, e.g., the effect of local electrically conductive paths shown to dominate the temperature‐dependent piezoelectric response of BFO,^[^ ^32^ ^]^ which is the major component of all the investigated BFO–BTO samples. In addition, they utilized a single BFO–BTO composition (30 mol% BTO) that consisted of typical core–shell chemical inhomogeneities and elevated electrical conductivity, all of which can severely impact the piezoelectric behavior of the ceramics.

From the fundamental point of view, several questions related to the piezoelectric response of lead‐free ferroelectrics with relaxor character remain unanswered and require further investigation. In particular, a specific mechanism(s) through which the configured “composite” domain structure acts to positively enhance the material properties are yet to be determined. An interesting question is whether the previously proposed hysteretic and nonlinear cascade‐like nanodomain‐wall motion inside a hierarchical domain structure, characteristic for the disordered relaxor phase of monoclinic PMN–PT,^[^ ^10^, ^33^ ^]^ could similarly contribute to the observed temperature‐enhancement of d_33_ in BFO–BTO. All these issues are critical to the understanding of the piezoelectric performance of lead‐free relaxor ferroelectrics but cannot be addressed without a proper experimental approach involving, e.g., harmonic analysis over a broad temperature and driving‐field conditions (frequency and field magnitude), as previously demonstrated for the case of PMN–PT.^[^ ^10^, ^34^ ^]^

In this work, we elaborate in detail the high‐temperature piezoelectric response of the BFO–BTO system over a wide compositional range (0–50% BTO), utilizing high‐quality ceramic samples with no core–shell inhomogeneities, negligible amounts of secondary phases, and controlled electrical conductivity. We demonstrate for the first time an unprecedented converse piezoelectric coefficient of 1200 pm V^−1^ achieved at temperatures exceeding 300 °C. Despite the fact that the response is confined to elevated temperatures, we nevertheless reveal the potential of the BFO–BTO system in exhibiting piezoelectric response comparable to the best room‐temperature d_33_ values of Sm‐doped PMN–PT systems reported in the literature.^[^ ^3^ ^]^ Based on a systematic isothermal nonlinear piezoelectric harmonic analysis in a wide temperature range (25–325 °C), electric‐field range (0.1–4.8 kV cm^−1^) and frequency range (0.03–100 Hz), combined with temperature‐dependent dynamic piezoelectric measurements, we reveal a distinct hysteretic and nonlinear piezoelectric response reminiscent to that characteristic for lead‐based relaxor ferroelectrics, such as PMN–PT, Pb(Fe_0.5_Nb_0.5_)O_3_ (PFN) and Pb(Sc_0.5_Nb_0.5_)O_3_ (PSN).^[^ ^10^ ^]^ Further analyses focused on the compositionally dependent relaxor disorder, local electrically conductive paths, and temperature‐dependent domain structure enabled us to identify and quantify the multiple contributions to the piezoelectric response of BFO–BTO. These multiple factors originating from the combined effects of relaxor disorder and electrical conductivity work together to enhance the overall piezoelectric response, providing valuable insights into the complex structure‐property relationships of BFO–BTO piezoelectric ceramic system.

## 2 Results and Discussion

### 2.1 Phase Composition, Dielectric, Piezoelectric, and Ferroelectric Properties of the BFO‐xBTO Compositional Series

**Figure 1:**
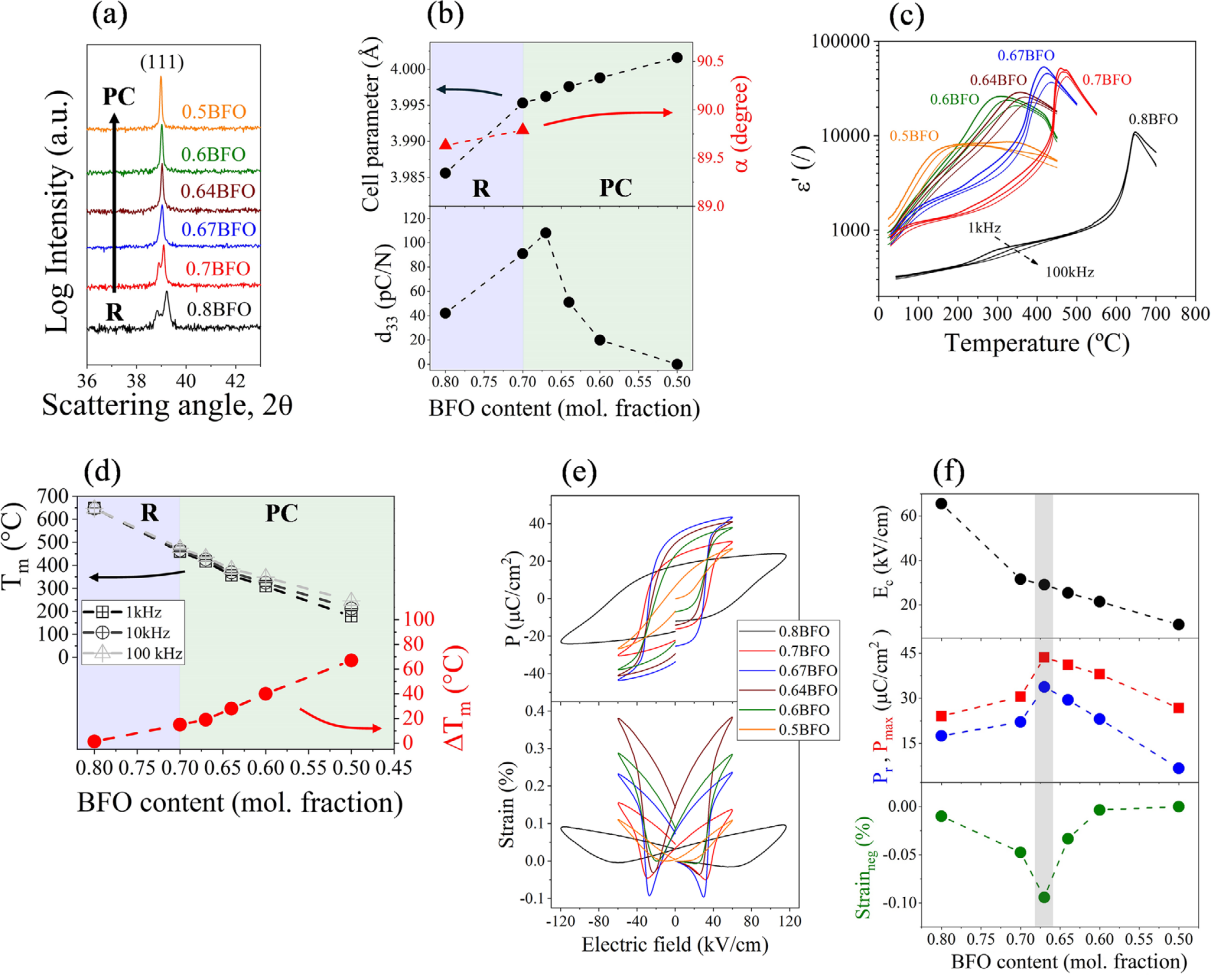
a) X‐ray diffraction (XRD) patterns of BFO–BTO compositional series highlighting the (111)_pc_ diagnostic peak. The arrow indicates the compositionally induced transition from rhombohedral (R) to pseudocubic (PC) symmetry. b) The upper panel shows the cell parameter (black dots) and the rhombohedral distortion angle (α, red dots) as a function of BFO content obtained from Rietveld refinement analysis. The lower panel shows the evolution of the piezoelectric d_33_ coefficient as a function of BFO content. The violet color indicates the rhombohedral (R) side of the phase diagram, while the green color indicates the pseudocubic (PC) side. c) Temperature dependence of dielectric permittivity (ɛ′) measured at 1, 10, and 100 kHz during heating of BFO–BTO series. d) Temperature of the maximum of the dielectric ɛ′ peak (T_m_) determined at 1, 10, and 100 kHz and the difference between T_m_ measured at 1 and 100 kHz (ΔT_m_) as a function of BFO content. The same color coding is used as in panel (b) to indicate the rhombohedral and pseudocubic sides. e) Polarization (P) and strain hysteresis loops of BFO–BTO series and f) extracted parameters from the hysteresis loops (coercive field E_c_, remanent polarization P_r_, maximum polarization P_max_ and negative strain Strain_neg_) as a function of BFO content.

To provide a comprehensive compositional overview of the BFO–BTO material system, we begin by analyzing the average crystal structure (**Figure** **1**a,b), the dielectric relaxor behavior (Figure 1c,d), and the ferroelectric properties (Figure 1e,f). Starting from the average crystal structure analysis (Figure 1a), a structural transition from a rhombohedral (R) to a pseudocubic (PC) symmetry is observed with increasing BTO content, confirmed by the evolution of the rhombohedral split peak into a single cubic‐like (111) peak (see arrow). Rietveld refinement analysis (details provided in Figures S1–S3, Supporting Information) reveals that the structural transition from rhombohedral (*R3c* space group) to pseudocubic (*Pm‐3m* space group) symmetry occurs at BTO contents exceeding ≈30 mol %, similarly as reported earlier (see, e.g., Ref.^[^ ^35^ ^]^). The incorporation of BTO into BFO leads to a decrease of the rhombohedral distortion (rhombohedral distortion angle* α* approaching 90°, see red curve in the upper plot of Figure 1b) and to an increase in the cell parameter, from 3.9856 for 0.8BFO to 4.0016 Å for 0.5BFO (black curve in the upper plot of Figure 1b). This latter observation can be rationalized by comparing the ionic radii of the elements in which case the radius of Ba^2+^ ions (1.61 Å) on the A‐sites is larger than that of Bi^3+^ ions (1.31 Å), whereas Fe^3+^ (0.645 Å) and Ti^4+^ (0.605 Å) ions on the B‐sites have more similar radii (12‐fold coordination for A‐site and 6‐fold coordination for B‐site elements are considered).^[^ ^20^ ^]^ The structural changes can be therefore associated with the larger Ba^2+^ ions incorporated at the A sites, as discussed previously.^[^ ^19^ ^]^

The 0.67BFO composition has been the subject of continuing discussion in the literature, as it occupies a transitional region between the rhombohedral and pseudocubic phases. Researchers have utilized diverse models in an attempt to elucidate the structural characteristics of this particular composition, including the pseudo‐cubic, rhombohedral symmetry, and the coexistence of these two phases.^[^ ^13^, ^21^, ^36^, ^37^ ^]^ It is often disregarded that the phase composition may also be linked to the chemical segregation effects, often referred to in the literature as the core–shell microstructure, where phase co‐existence may be linked to the presence of BFO‐ and BTO‐rich inhomogeneities.^[^ ^21^, ^37^, ^38^ ^]^ Furthermore, the 0.67BFO composition is found to be particularly intriguing as it exhibits a maximum in the piezoelectric d_33_ coefficient (see Figure 1b, bottom plot), although it appears to show a cubic‐like average structure (see blue‐green crossover in Figure 1b). The 0.67BFO composition consists of a nonergodic‐like relaxor phase with a pseudocubic average structure but with a local symmetry clearly deviating from cubic,^[^ ^39^ ^]^ as further confirmed by synchrotron analysis (see details in Figure S4, Supporting Information). As directly observed by in situ diffraction analysis in a similar relaxor‐ferroelectric ceramic system, i.e., BiFeO_3_–SrTiO_3_,^[^ ^40^ ^]^ the application of an electric field results in the appearance of a rhombohedral‐like average distortion, consistent with a field‐induced transition from a non‐ergodic to a ferroelectric phase. A transition from a pseudocubic to a ferroelectric phase induced by the application of an electric field, accompanied by a transformation from a disordered nanoscale to long‐range‐ordered ferroelectric‐like domains, was also consistently observed in BFO–BTO ceramics.^[^ ^17^, ^22^ ^]^

The relaxor features as a function of BFO content, as observed from dielectric spectroscopy analysis, are further explored in the following panels. Figure 1c shows the dependence of the real part of dielectric permittivity (ɛ′) with temperature for the different BFO–BTO compositions, while Figure 1d shows the analyses of the evolution of the temperature of the permittivity maximum (*T_m_*) and the calculated temperature difference of *T_m_* measured at 1 and 100 kHz (Δ*T_m_*), which is commonly employed to quantify the relaxor characteristics. The data clearly demonstrate the shift of the frequency‐dispersed ɛ′ peaks toward lower temperatures, accompanied by a significant broadening and increased frequency dispersion of the permittivity maximum, as the BFO concentration decreases (Figure 1c). Quantitative analysis reveals that the dispersion, expressed as Δ*T_m_*, ranges from ≈1 °C for 0.8BFO to ≈67 °C for 0.5BFO (Figure 1d, red curve), confirming the strong disorder induced by BTO addition, most likely originating from local fluctuations of charge (ionic valence) and strain (ionic size) on both A and B perovskite sites.^[^ ^29^, ^41^ ^]^ It is worth noting in Figure 1d that the slope of the *ΔT_m_* evolution as a function of BFO changes, becoming steeper as soon as the system enters the pseudocubic region of the phase diagram, i.e. above ≈30 mol% BTO (see blue/green crossover in Figure 1d denoting the rhombohedral, *R*, and pseudocubic, *PC*, regions). The results therefore demonstrate that the relaxor character increases with decreasing BFO content, particularly at concentrations above ≈30 mol% BTO (i.e., in the PC region).

Another important point that we analyze is that the addition of BTO to BFO leads to a decrease of *T_m_* (Figure 1d, black curves), meaning that it drives the nonergodic‐to‐ergodic transition toward room temperature, an effect analogous to that in PMN–PT when compositionally approaching the PMN ergodic relaxor end‐member.^[^ ^10^ ^]^ To investigate this hypothesis, we performed a Vogel–Fulcher (V–F) analysis on the experimental dielectric data of both 0.67BFO and 0.5BFO samples. The results, presented in detail in Figure S5 (Supporting Information), indicate the approximate freezing temperatures of 370 and 23 °C for the 0.67BFO and 0.5BFO samples, respectively, with similar activation energies *E_a_* of ≈0.06 eV (for comparison, *E_a_* related to freezing phenomenon in the canonical PMN relaxor is on the level of ≈0.04 eV).^[^ ^42^ ^]^ Therefore, the addition of BTO to BFO not only enhances the relaxor disorder (increased dispersion Δ*T_m_*, Figure 1d) but also increases the stability of the ergodic phase closer to room temperature (decreased *T_m_*, Figure 1d, and freezing temperature, Figure S5, Supporting Information). Both observations are consistent with previous studies.^[^ ^35^ ^]^

The enhanced relaxor disorder by BTO addition is also reflected in the ferroelectric properties, as can be inferred from Figure 1e, which shows the polarization (*P*) and strain electric‐field hysteresis loops. Interestingly, none of the P‐E loops show an obvious pinched shape as observed in the end‐member BFO featured by a strong hardening character.^[^ ^43^ ^]^ By analyzing in detail the hysteresis‐loop parameters, including the coercive field *E_c_*, remanent polarization *P_r_*, maximum polarization *P_max_* and negative strain value *Strain_neg_* (Figure 1f), it emerges that *E_c_* decreases as a function of the BTO concentration, thus demonstrating a progressive “softening” behavior in terms of domain switching. Moreover, the maximum values of *P_r_*, *P_max_* and *Strain_neg_* are achieved at the 0.67BFO composition, which is consistent with the maximum d_33_ value (Figure 1b).

The analysis presented in Figure 1 clearly demonstrates that the ferroelectric and piezoelectric properties are maximized at the 0.67BFO composition. Subsequently, we investigate the potential mechanisms underlying the observed decrease in properties at both compositional sides (BFO‐rich and BTO‐rich regions) relative to the 0.67BFO composition. The data confirm an enhanced relaxor disorder as BTO content is added continuously from 0.8BFO to 0.5BFO (see Δ*T_m_* in Figure 1d). Despite this trend, a decline in the piezoelectric d_33_ coefficient (Figure 1b) and ferroelectric properties (*P_r_*, *P_max_* and *Strain_neg_*; Figure 1f) is observed in compositions richer in BTO relative to 0.67BFO (i.e., 0.64BFO, 0.6BFO, and 0.5BFO). The loss of the ferroelectric character and, consequently, piezoelectric response, can be attributed to the gradual stabilization of the dynamic ergodic relaxor state closer to room temperature, particularly at compositions richer in BTO relative to the 0.67BFO at which maxima are observed. This transition is evidenced by two key observations. First is the evolution of P‐E hysteresis loops where the loops progressively narrow with BTO addition (Figure 1e; see, e.g., the slim loop of 0.5BFO extreme composition) and where the remanent polarization (*P_r_*) and negative strain (both related to the permanent ferroelectric domain‐switched state) diminishes toward zero as the BFO content is decreased below 0.67BFO (Figure 1f). The second evidence is the shift of the V‐F freezing temperature toward room temperature in the limiting 0.5BFO composition (see Figure S5, Supporting Information). Both arguments are consistent with previous studies on BFO–xBTO compositional series.^[^ ^35^ ^]^ On the other hand, the decrease of the piezoelectric and ferroelectric properties in BFO‐richer compositions relative to 0.67BFO, i.e., 0.7BFO and 0.8BFO (see d_33_ in Figure 1b, and *P_r_*, *Strain_neg_* in Figure 1f) is more difficult to understand and may involve multiple mechanisms. Limited by the current data and understanding, we may consider two aspects. The first is related to the so‐called “hardening” behavior of unmodified BFO, eventually diminishing compositionally as BTO is added to the solid solution. At least a part of the hardening behavior of BFO is, in fact, related to its p‐type conductivity and associated Fe^4+^ pinning centers concentrated at the domain walls, ^[^ ^44^ ^]^ which may be reduced by adding BTO, similarly as suggested in the case of BFO–(K_0.5_Bi_0.5_)TiO_3_ (BFO–KBT) system.^[^ ^45^ ^]^ Even in the hypothetical absence of this scenario, i.e., reduced amount of pinning centers, the increased lattice disorder induced in BFO by chemical modification with BTO may itself lead to a more disordered domain‐wall pinning potential and thus ferroelectric and piezoelectric “softening” effects,^[^ ^45^ ^]^ similar to those occurring in PZT.^[^ ^46^ ^]^ Second, one has to consider the compositionally induced relaxor behavior and the associated relaxor “softening” effects, as discovered in PMN–PT,^[^ ^10^ ^]^ where the nanodomain structure plays the key role. All these scenarios are consistent with a pronounced decrease of *E_c_* and concurrent increase of *P_r_*, *P_max_* and *Strain_neg_* as well as with increased *d*_33_ coefficient observed from 0.8BFO toward 0.67BFO composition (see Figure 1b,f). Although not clear at the present stage, if these scenarios are correct, the maximum in the piezoelectric response of 0.67BFO may not necessarily arise due to MPB‐like effects, which seems to be commonly accepted in the literature.

### 2.2 High‐Temperature Piezoelectric and Dielectric Response

**Figure 2:**
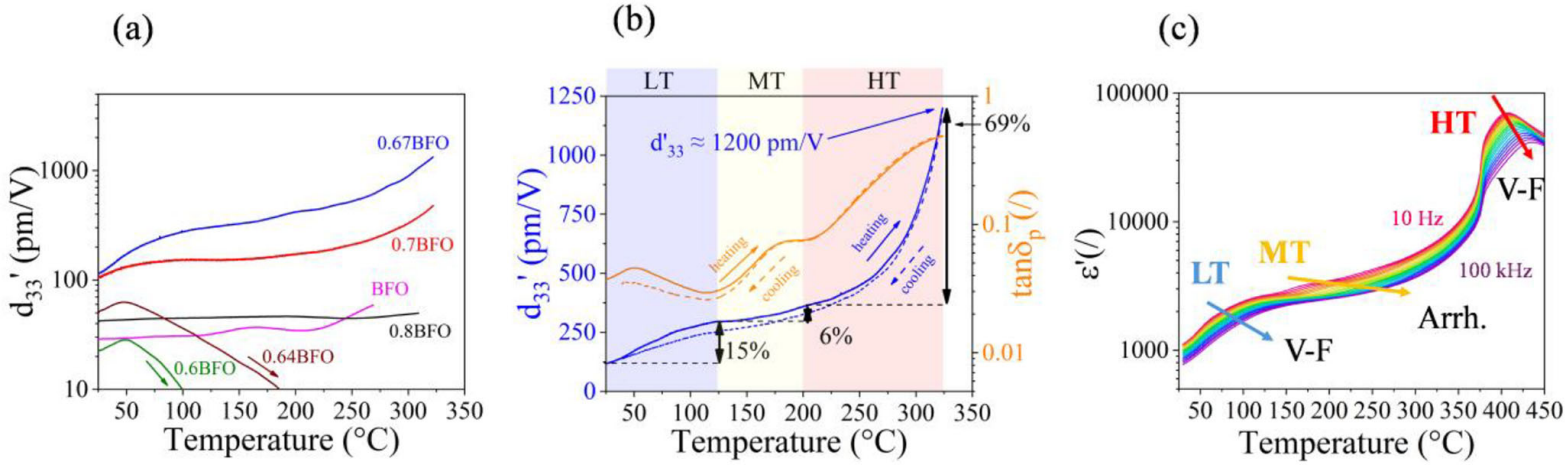
a) Temperature dependence of piezoelectric $d_{33}^{\prime }$ coefficient for BFO–BTO compositional series. b) Evolution of the piezoelectric $d_{33}^{\prime }$ coefficient (blue curves) and tangent of the piezoelectric phase angle *tan*δ_*p*_ (orange curves) during the heating (full curves) and cooling cycle (dashed curves) for the 0.67BFO sample. Distinct chromatic designations were assigned to each identified region (low‐temperature – LT, middle‐temperature – MT, and high‐temperature – HT) to facilitate differentiation and analysis. For each of the stages, the corresponding relative contribution to the total $d_{33}^{\prime }$ is indicated in percentages. c) Temperature dependence of dielectric permittivity (ɛ′) measured from 10 Hz to 100 kHz for 0.67BFO sample showing different types of thermal activation for each identified dispersion process (Arrh. – Arrhenius type; V‐F – Vogel‐Fulcher type).

After the introduction of the basic structural and relaxor characteristics of the synthesized BFO–BTO compositional series, we next examine the piezoelectric properties as a function of temperature. **Figure** **2**a shows the temperature dependence of the real part of the piezoelectric $d_{33}^{\prime }$ coefficient for each composition analyzed (note the logarithmic scale). Upon initial examination, it is immediately evident that the 0.67BFO sample (blue line) exhibits the highest piezoelectric response in the entire temperature range. Notably, the piezoelectric coefficient increases significantly, surpassing 1000 pm V^−1^ at temperatures exceeding 300 °C. Qualitatively speaking, the temperature trend of 0.67BFO follows a non‐monotonic, multiple‐step‐like $d_{33}^{\prime }$ behavior. As shown later, it is this behavior that embeds the information on the multiple origins of the exceedingly large $d_{33}^{\prime }$ response. The 0.7BFO sample (red curve) exhibits a similar temperature trend to that of the 0.67BFO sample, albeit consistently displaying a lower coefficient. In strong contrast to these 0.7BFO and 0.67BFO compositions, the BTO‐richer samples, i.e., 0.64BFO (brown curve) and 0.6BFO (green curve), demonstrate a completely different behavior characterized by a maximum in the piezoelectric coefficient. Initially, the piezoelectric coefficient exhibits an increasing trend, similar to that observed for 0.7BFO and 0.67BO samples, however, this pattern undergoes a reversal at ≈50 °C, after which the piezoelectric coefficient begins to decline, which is due to thermal depoling (confirmed by zero piezoelectric coefficient after the measurements).^[^ ^17^ ^]^ This depoling can be attributed to the stabilization of the ergodic phase with dynamic polar nanoregions appearing closer to room temperature in BTO‐richer compositions, as discussed in the previous section. Finally, the BFO‐richest compositions, i.e., BFO (pink curve) and 0.8BFO (black curve), exhibit a much weaker temperature dependence of the piezoelectric coefficient when compared to other compositions. It will be shown at a later stage that the reason lies in the absence of a sufficient relaxor disorder in these BFO‐rich compositions; consequently, this leads to a lack of the temperature‐driven evolution of the domain structure into a relaxor‐featured hierarchical arrangement of nanodomains inside larger domains, as recently observed in monoclinic PMN‐PT ceramics.^[^ ^10^ ^]^

Focusing on the sample that exhibited the highest piezoelectric response, i.e. 0.67BFO, Figure 2b illustrates the evolution of the piezoelectric $d_{33}^{\prime }$ coefficient (blue curves) and piezoelectric *tan*δ_*p*_ (orange curves) during the heating (full curves) and cooling cycle (dashed curves). At first glance, we can observe that the heating and cooling curves are very similar, which suggests that the sample does not depole even when subjected to a maximum temperature of ≈325 °C. We note that this temperature was used as the maximum measurement temperature; the eventual depoling behavior above 325 °C was not studied. Nevertheless, this stable poled state provides great promises to the BFO–BTO ceramics system in terms of high‐temperature piezoelectric applications. Furthermore, the longitudinal piezoelectric coefficient reaches a maximum value of 1200 pm V^−1^ at ≈325 °C, which is comparable to the room‐temperature *d*_33_ values of the best‐performing PMN–PT relaxor ferroelectric ceramics reported in the literature.^[^ ^3^, ^47^, ^48^ ^]^ It is important to emphasize that Figure 2b plots the small‐signal piezoelectric coefficient, as the sample was driven using a sub‐coercive electric field in the whole temperature range (see details on the evolution of *E_c_* with temperature in Figure S6, Supporting Information).

Based on the peaks in *tan*δ_*p*_ observed in Figure 2b, accompanied by incremental (step‐like) anomalies in $d_{33}^{\prime }$, we can identify three distinct stages in the temperature evolution of the piezoelectric response of 0.67BFO, which are denoted here as low temperature (LT), medium temperature (MT), and high temperature (HT), each indicated by different colors (blue, yellow and red, respectively). It is reasonable to think that each of the three stages is linked to a particular microscopic mechanism, contributing to the d_33_ coefficient with increasing temperature, which implies that the large coefficient of 1200 pm V^−1^ cannot be explained using a single mechanism. The first stage, i.e., from room temperature to ≈125 °C (LT), accounts for 15% of the total piezoelectric coefficient (i.e., 1200 pm V^−1^). The second stage (MT), from ≈125 to 200 °C, contributes a lesser amount, only 6%. The final stage (HT) demonstrates by far the largest piezoelectric contribution, i.e., 69% of the total piezoelectric response. This quantitative assessment does not necessarily mean that the different contributions are additive, as they may mutually interact, giving rise to emergent phenomena (see, e.g., Ref.^[^ ^11^ ^]^). However, the analysis reveals the quantitative importance of the HT contribution. Finally, we note that the temperature‐dependent piezoelectric contributions show relaxational behavior as confirmed by isothermal frequency‐dependent $d_{33}^{\prime }$ measurements (see Figure S7, Supporting Information). Some important differences arise from this analysis, e.g., while the thermally activated piezoelectric relaxation in the MT region has little or no dependence on the applied field, the HT relaxation is strongly activated by the electric field magnitude.

With the aim of further investigating the electrical characteristics of the three stages of piezoelectric enhancements, we analyzed the temperature dependence of the permittivity of poled 0.67BFO over a wide range of frequencies (from 10 Hz to 100 kHz) and by performing Vogel‐Fulcher (V‐F) fitting analysis. The dielectric analysis is justified because the three contributions (LT, MT, HT), accompanied by distinct peaks in the piezoelectric phase angle (*tan*δ_*p*_; Figure 2b, orange curves), can be also identified by the same peaks in the dielectric loss (*tan*δ_*d*_; see Figure S8, Supporting Information). A graphical depiction of the results can be found in Figure 2c, while for further details of the analysis refer to Figure S9 (Supporting Information). The first stage (LT) presents a V‐F‐type relaxation, the second (MT) instead is best represented by a thermal activation of the Arrhenius‐type, while the third is again of the V‐F‐type, revealing a very complex sequence of thermally activated relaxational processes. A dielectric relaxation closely obeying the V‐F relation can be generally treated in the frame of relaxor behavior, specifically, it can be attributed to the dynamic contribution and freezing of polar nanoregions as the temperature is decreased.^[^ ^42^ ^]^ Subsequent microscopy analysis, shown later in this section, in fact, reveals that the first relaxation (LT) can be attributed to the re‐entrant relaxor effect,^[^ ^49^, ^50^ ^]^ while the third (HT) is likely related to the interface dynamics inside hierarchically structured nanodomains, arising as well from the relaxor disorder.^[^ ^10^, ^34^ ^]^ Instead, the MT relaxation is different in nature and indicates a thermally activated conductivity process, most likely of a Maxwell‐Wagner (M‐W) origin, as recently suggested for Nb‐doped BFO–BTO.^[^ ^51^ ^]^ The results of the fitting analysis for each region are collected in **Table** **1** (for additional details, refer to Figure S9, Supporting Information). Note that the Arrhenius‐like thermally activated relaxation is clearly distinguished from the two V‐F‐like relaxations in its higher activation energy E_a_ (1.13 eV for MT vs 0.025 eV and 0.065 eV for LT and HT stages, respectively).

**Table 1:** Parameters obtained from Vogel‐Fulcher (V‐F) and Arrhenius fitting analyses of the dielectric response of 0.67BFO samples across different temperature stages (LT, MT, HT). E_a_ and T_f_ denote the activation energy and freezing temperature, respectively.

Stage	Model	E_a_ [eV]	T_f_ [ °C]
LT	V‐F	0.025	61
MT	Arrhenius	1.13	/
HT	V‐F	0.065	370

The BFO–BTO solid solution is composed of the BFO end member that is known to exhibit elevated electrical conductivity and typical dielectric and piezoelectric M‐W relaxational effects arising from local variations in the electrical conductivity.^[^ ^32^, ^52^ ^]^ Possible M‐W effects in BFO–BTO are further elaborated by the analysis shown in Figure S10a (Supporting Information) where the temperature‐dependent piezoelectric response across the three relaxational stages is more closely compared between the 0.8BFO, 0.7BFO, and 0.67BFO samples. The most relevant for the interpretation are two features identified in the 0.8BFO sample within the MT stage: i) a negative piezoelectric phase angle (refer to Figure S10a, Supporting Information) and ii) local variations in the electrical conductivity close to grain boundary areas (see conductive atomic‐force microscopy analysis in Figure S10b, Supporting Information). Both these observations consistently point toward the M‐W piezoelectric effect, similarly as suggested for BFO.^[^ ^52^ ^]^ It is not impossible that BTO‐richer compositions, like 0.67BFO, may exhibit some residual M‐W effects originating from the BFO component, which is consistent with the Arrhenius‐type thermally activated relaxation in the MT region (Figure 2c). In fact, the same type of dielectric relaxation that is thermally activated according to Arrhenius law is observed in the 0.8BFO sample, characterized by the same activation energy (≈1 eV) as that found for the MT relaxation of 0.67BFO (refer to Figure S10c,d and Table S3 for details, Supporting Information).

### 2.3 Temperature‐ and Electric‐Field‐Dependent Nonlinear Piezoelectric Harmonic Response

**Figure 3:**
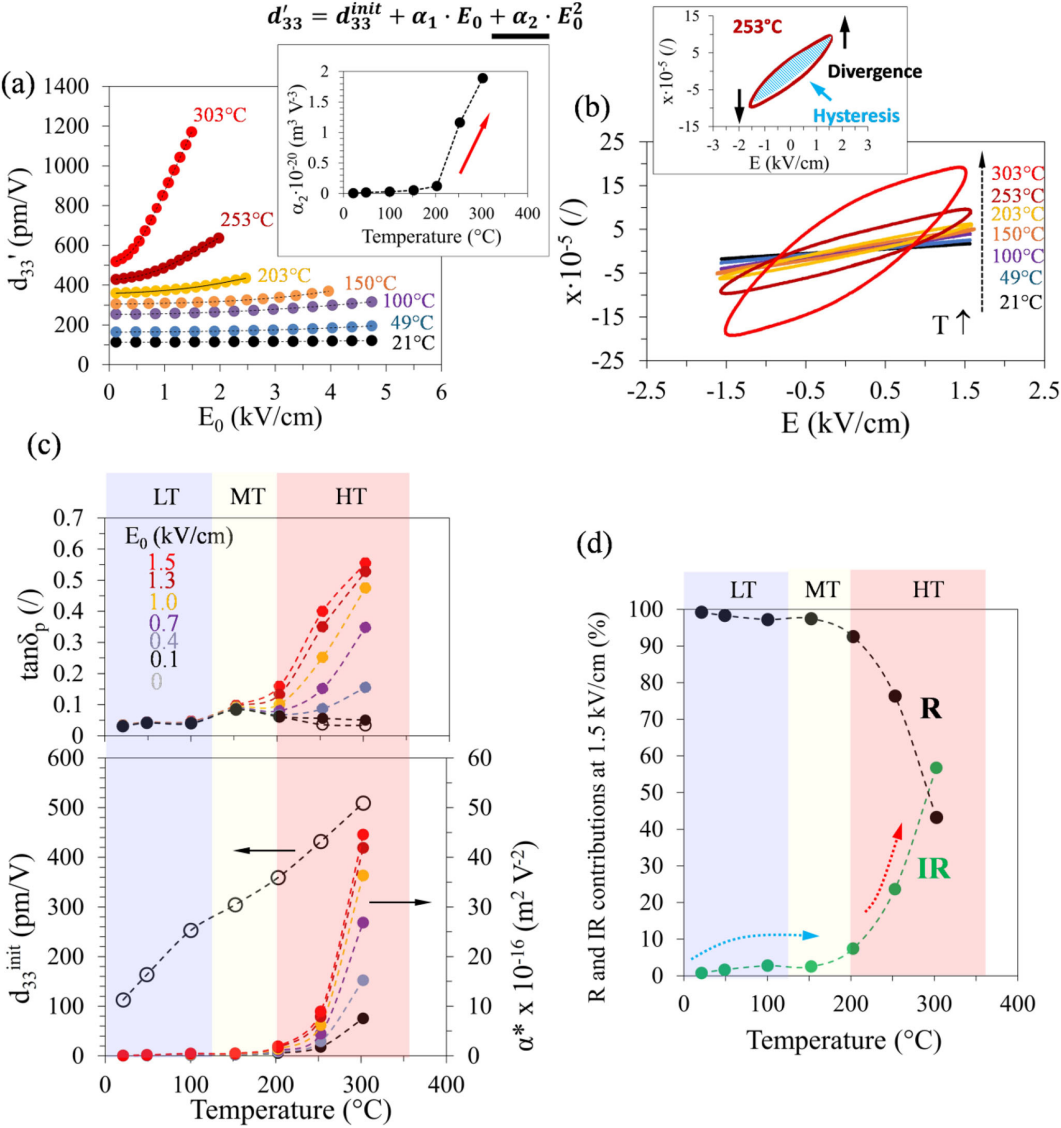
a) Converse piezoelectric $d_{33}^{\prime }$ coefficient as a function of electric field amplitude (*E*_0_) for 0.67BFO at different temperatures. The *E*_0_‐dependent $d_{33}^{\prime }$ data were fitted with a second‐order polynomial, and the resulting equation and quadratic coefficient (α_2_) plotted against temperature are shown in the inset. b) Piezoelectric hysteresis loops (strain *x* vs field *E*) of 0.67BFO sample at different temperatures. The inset shows details of the loop shape at 253 °C, i.e., the large hysteresis and divergent‐like loop deformation. c) Temperature dependence of the tangent of the piezoelectric phase angle *tan*δ_*p*_ (upper plot) and of $d_{33}^{init}$ and α* coefficients (lower plot) of 0.67BFO ceramics extracted for different *E*_0_. d) Relative fractional contributions of the reversible (R) and irreversible (IR) response to the total $d_{33}^{\prime }$ measured at *E*_0_= 1.5 kV cm^−1^. The individual stages (LT, MT, HT) are denoted with colored bands in panels (c) and d). The blue and red arrows in panel (d) highlight the plateau‐like and exponential‐like temperature trends in the irreversible response, respectively.

To investigate the origins of the different contributions in the temperature‐dependent piezoelectric response of 0.67BFO, we add another parameter in the analysis, i.e., the electric field magnitude. **Figure** **3**a illustrates the electric‐field dependence of $d_{33}^{\prime }$ at various temperatures up to ≈300 °C (isotherms; see Experimental Section), while Figure 3b shows the piezoelectric hysteresis at the same temperatures and fixed electric field amplitude of 1.5 kV cm^−1^. Inspection of Figure 3a reveals a significant increase in the piezoelectric coefficient as a function of temperature, which is consistent with the results of the dynamic experiments (see Figure 2b). More importantly, the nonlinear response, i.e., electric‐field‐amplitude (*E*_0_) dependency of $d_{33}^{\prime }$, increases significantly with increasing temperature (see change of $d_{33}^{\prime }-E_{0}$ slope in Figure 3a). In addition, a qualitative change in the $d_{33}^{\prime }-E_{0}$ relation is observed, becoming strongly superlinear (up‐curved) when approaching and exceeding 200 °C. In order to quantitatively illustrate this phenomenon, we fitted the $d_{33}^{\prime }-E_{0}$ data at each temperature using a second‐order polynomial function and plotted the quadratic polynomial coefficient (α_2_) obtained from these fittings in the inset in Figure 3a. The results confirm an onset of a strong $d_{33}^{\prime }-E_{0}$ superlinearity (upcurving) above 200 °C (red arrow in Figure 3a inset), i.e., in the HT stage (red area in Figure 2b) where α_2_ increases abruptly. When comparing these data with the hysteresis loops in Figure 3b, it becomes evident that the superlinear behavior is accompanied by distinct qualitative changes in the hysteresis‐loop shape above 200 °C. The changes can be summarized by the appearance of exceedingly large hysteresis (i.e., large phase angle; see loop area abruptly increasing above 200 °C) and divergent‐like deformation (up‐ and down‐curving of the tips of the loop; see inset of Figure 3b for the case of 253 °C). These two features alongside the $d_{33}^{\prime }-E_{0}$ superlinearity represent the fingerprint of the nonlinear hysteretic response discovered in lead‐based relaxor ferroelectrics, including PMN–PT, PSN, and PFN,^[^ ^10^ ^]^ which are associated with the dynamic response of domain walls in these disordered relaxor‐based materials. Details of this contribution, which is present also in BFO–BTO above 200 °C (HT stage), will be discussed in detail later in this section.

We proceed with the analysis on the most active 0.67BFO sample by applying the Rayleigh law, which allows us to separate the reversible and irreversible contributions from the total piezoelectric response. For further details about the method and symbol definitions, see Section S9 (Supporting Information). The coefficient $d_{33}^{init}$, which represents the intercept of the $d_{33}^{\prime }$ vs *E*_0_ curve at field zero, contains both lattice and non‐lattice reversible contributions, while α*, which represents the slope of $d_{33}^{\prime }$ vs E_0_ curve, is assumed to comprise only irreversible non‐lattice contributions. In the case of lattice contribution, we are referring to the so‐called “intrinsic” effect, which is mainly governed by cation displacement within a single domain under an applied electric field; conversely, with the non‐lattice effect, often called “extrinsic,” we are referring to the irreversible domain‐wall (DW) or interphase‐boundary displacements (in case of the presence of multiple phases, like in MPB compositions).^[^ ^53^, ^54^ ^]^

The temperature dependence of $d_{33}^{init}$ and α* coefficient (evaluated at different *E*_0_) is plotted in the lower part of Figure 3c. The piezoelectric phase angle *tan*δ_*p*_ is added for comparison in the upper part of the panel. Starting with the analysis of the irreversible coefficient α*, it is evident that the response both in the LT and MT regions is weakly nonlinear, i.e., characterized by relatively low α* values, i.e., in the range 0.02–0.5∙10^−16^ m^2^V^−2^ (taken as extremes in the temperature and electric‐field interval of 25–150 °C and 0.1–1.5 kV cm^−1^, respectively). For comparison, at room temperature, a morphotropic soft PZT would achieve at least an order of magnitude larger irreversible coefficients (level of 15∙10^−16^ m^2^V^−2^),^[^ ^34^ ^]^ while a very soft material, such as Sm‐doped PMN–PT, can even reach two‐to‐three orders of magnitude higher α* (up to 145∙10^−16^ m^2^V^−2^), also at room temperature.^[^ ^11^ ^]^ A transition from weakly to strongly nonlinear “soft”‐like response in 0.67BFO is clearly observed in the HT region, where α* reaches values of up to 45∙10^−16^ m^2^V^−2^. Note that in this HT region, the α* coefficient becomes field‐dependent (see the increase of α* with *E*_0_ in Figure 3c above 200 °C, bottom plot), which essentially means that the *d*_33_ − *E*_0_ relation becomes superlinear as discussed earlier (Figure 3a, inset). The enhancement in the nonlinearity in the HT region is accompanied by large and field‐dependent *tan*δ_*p*_ (Figure 3c, upper plot), suggesting an enhanced nonlinear hysteretic response, commonly attributed to irreversible DW displacements.^[^ ^55^ ^]^

In contrast to the irreversible contribution, the reversible coefficient ($d_{33}^{init}$ in Figure 3c) increases with temperature quasi‐monotonically until reaching the maximum value of 509 pm V^−1^ at 303 °C. A slight deviation from a linear trend, seen as a plateau‐like feature, can be observed in $d_{33}^{init}$ at ≈150 °C, which is coupled to a peak in *tan*δ_*p*_ at the same temperature (Figure 3c, upper graph). Both these features in the MT region are consistent with the observations from the dynamic experiment (see the weak plateau‐like feature in $d_{33}^{\prime }$ and the associated peak in *tanδ_p_* in MT region in Figure 2b).

In addition to the two Rayleigh coefficients, it is insightful to extract the fractional contributions of the reversible and irreversible response (further details are explained in Section S9, Supporting Information). Figure 3d displays the two contributions at 1.5 kV cm^−1^ as a function of temperature. Focusing on the IR contribution (green line), after an initial slight increase to ≈3% at 100 °C, the irreversible component appears to reach a saturation level at ≈150 °C in the MT region (see blue arrow), before resuming a dramatic increase above 200 °C (see red arrow), which is when the sample enters the HT region. It is also important to note that the onset of the exponential increase in the irreversible component coincides with the onset of the dependence of the α* coefficient and *tanδ_p_* on the electric‐field amplitude (see Figure 3c). The data confirm that the piezoelectric response of 0.67BFO is mostly linear in the LT and MT regions, where the irreversible contribution is < 6%. In the HT region, however, the irreversible contribution increases sharply, reaching levels above 50%, thus becoming comparable to the reversible contribution.

**Figure 4:**
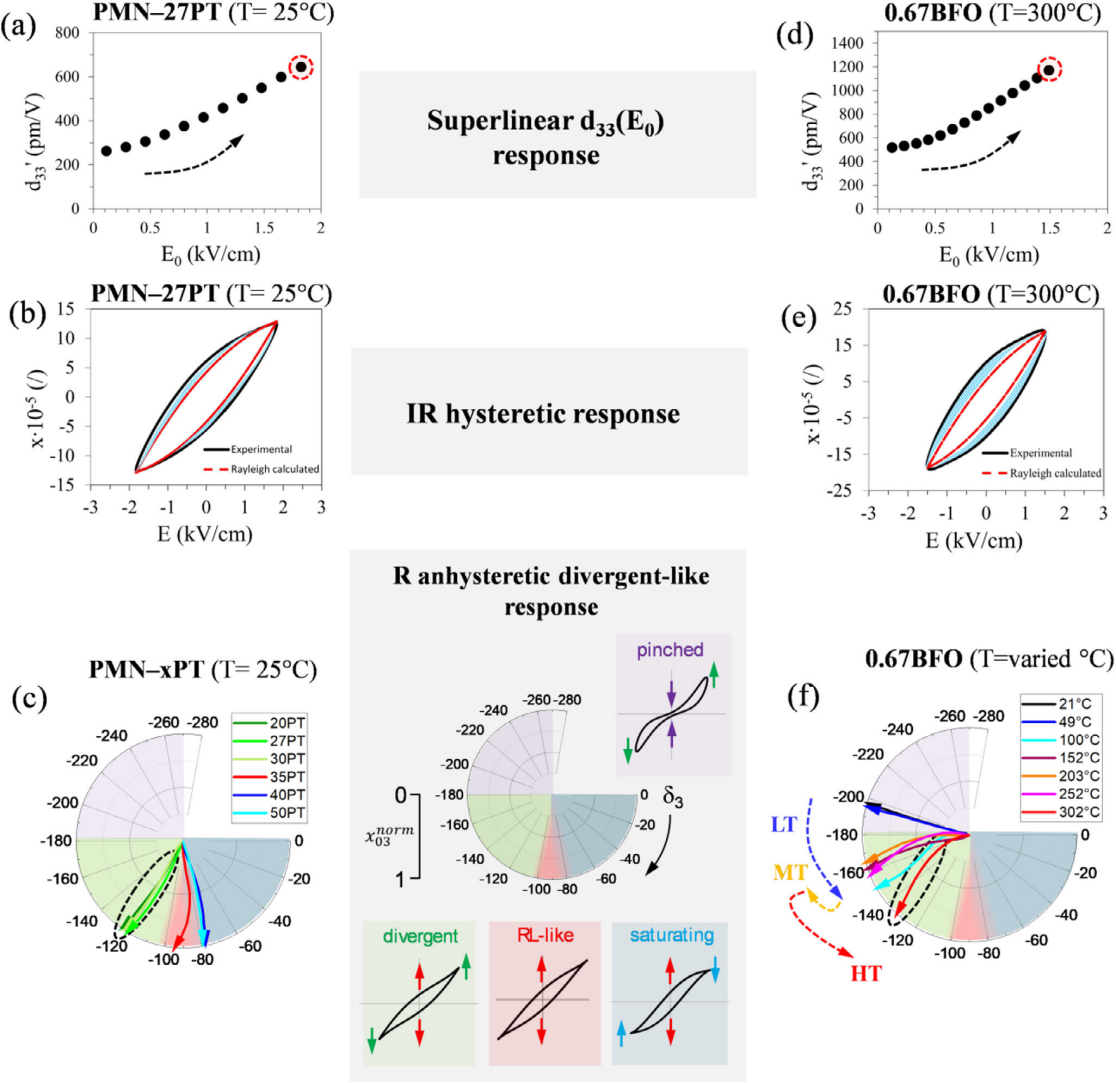
Comparison of the nonlinear piezoelectric response of PMN–27PT at room temperature and 0.67BFO at 300 °C, together with the compositional (PMN–xPT) and temperature (0.67BFO) evolution of the third‐harmonic response. a) Piezoelectric $d_{33}^{\prime }$ coefficient as a function of electric‐field amplitude, *E*_0_, and b) experimental piezoelectric hysteresis loop (black) (measured at the maximum field as depicted by the dashed‐red circle in panel (a)) and calculated Rayleigh law loop (red) of PMN–27PT at room temperature. The dashed blue region highlights the difference between the experimental and calculated loops (details of the calculation of the Rayleigh law loop are reported in Section S9, Supporting Information). c) Evolution of the third‐harmonic piezoelectric phase angle (δ_3_) across the PMN–xPT compositional series (20 ≤ x ≤ 50) represented in the phasor diagram (room‐temperature data; δ_3_ phasors are shown as a function of third‐harmonic piezoelectric strain amplitude normalized to the maximum value, $x_{03}^{norm}$, as it develops with increasing *E*_0_). The schematic phasor diagram (gray box) illustrates the different types of the third‐harmonic response, characterized by typical loop deformations (as indicated in loop schematics): i) pinched‐like (purple area), ii) divergent‐like (green area), iii) Rayleigh law‐like (red area) and iv) saturating‐like (blue area). d) Piezoelectric $d_{33}^{\prime }$ coefficient as a function of *E*_0_ and e) piezoelectric hysteresis loop (experimental – black, calculated – red) of 0.67BFO at 300 °C.) f) Phasor diagram showing the evolution of δ_3_ at different temperatures for 0.67BFO (the transitions between the LT, MT, and HT stages are noted with colored‐dashed arrows). The response of both PMN–27PT and 0.67BFO are characterized by the same superlinear $d_{33}^{\prime }-E_{0}$ behavior (panels a,d) and irreversible (IR) hysteresis that is larger than that predicted by Rayleigh law (dashed blue regions in panels b,e). Similarities in the δ_3_ behavior of the PMN‐rich compositions and 0.67BFO in the HT region are also observed (see circled phasors in panels c,f, both developing in the green quadrant). For additional details of the nonlinear harmonic piezoelectric response PMN–xPT ceramics, refer to Refs.[^[^ ^10^, ^34^ ^]^]

As anticipated, the nonlinear and hysteretic piezoelectric behavior of 0.67BFO observed in the HT region (> 200 °C) resembles that of lead‐based relaxor ferroelectrics.^[^ ^10^ ^]^ That response was found to be common for a broad range of monoclinic PMN–xPT compositions, i.e., 20 ≤ x ≤ 30, and was identified by its characteristics deviating from the Rayleigh law.^[^ ^10^, ^34^ ^]^ To illustrate this point we choose PMN–27PT as the representative composition and compare its room‐temperature piezoelectric behavior in **Figure** **4** to that of 0.67BFO at 300 °C. We underline three distinct nonlinear and higher harmonic responses that distinguish the response of monoclinic PMN–xPT: i) the superlinear $d_{33}^{\prime }-E_{0}$ dependency (see arrow in Figure 4a), deviating from the ideal linear relationship as predicted by Rayleigh law (see Section S9, Supporting Information), ii) the piezoelectric hysteresis that is systematically larger than that predicted by Rayleigh law equation (Equation S2, Supporting Information) using measured $d_{33}^{\prime }-E_{0}$ data (see blue shaded part in Figure 4b) and iii) the divergent‐like response described by the third‐harmonic phase angle appearing in the quadrant of the phasor diagram between –180° and –100° (see phasors surrounded by a dashed circle in the green area in the phasor diagram in Figure 4c), thus clearly deviating from that predicted by Rayleigh law, i.e., δ_3_ = –90° (see red area in the phasor diagram in Figure 4c and Section S9, Supporting Information). All three features of the nonlinear response also appear in the piezoelectric behavior of 0.67BFO at elevated temperatures (Figure 4d–f). Quantitative differences are obviously observed, e.g., relative to PMN–27PT, the larger deviation of the measured hysteresis in 0.67BFO with respect to  that predicted by Rayleigh law (see blue shaded area in Figure 4e) can arise due to greater losses owing to large thermal energy (300 °C).

The third harmonic piezoelectric response (Figure 4c,f) is worth further discussion. As explained in the study by Otoničar *et al.*,^[^ ^10^, ^34^ ^]^ the distinct nonlinear response emerges when the PMN–xPT composition becomes sufficiently rich in the relaxor PMN component to induce the particular dynamic response originating from the relaxor disorder. The reversible nonlinear anhysteretic component of this response, as it develops with composition (x in PMN–xPT), is illustrated in Figure 4c. PMN‐poorer tetragonal and MPB compositions with 35 ≤ x ≤ 50 show Rayleigh‐law‐like characteristics, seen by the phasors (colored arrows in Figure 4c) retained close to Rayleigh law predicted δ_3_ of –90° (i.e., close or inside the red triangle in the phasor diagram; see also loop schematic between panels c and f of Figure 4). As the PMN content is increased above the critical point (x < 35), a transition is observed in δ_3_, developing toward –120°, i.e., inside the green quadrant, where the divergent‐like response becomes significant (see PMN–20PT, PMN–27PT and PMN–30PT, dashed circle, and also loop schematic between panels c and f of Figure 4).

The evolution of the third harmonic response is also shown for 0.67BFO, this time as a function of temperature, with the transitions across the three (LT, MT, HT) stages noted with colored arrows (Figure 4f). Interestingly, at 21 °C, δ_3_ shows values close to –200°, thus staying in the purple quadrant between –180° and –270° where the loops exhibit a pinched‐like shape (see loop schematic between panels c and f of Figure 4). In the LT stage (follow the blue arrow), δ_3_ makes an abrupt turn toward –150° (see 100 °C in Figure 4f), effectively losing the pinched component and entering the green quadrant. While the origin of this loop opening from a pinched state during the LT re‐entrant relaxor transition is not entirely clear, it might be associated with multiple mechanisms, including i) the reversible ergodic‐relaxor‐to‐ferroelectric transition, disappearing at higher temperatures above the freezing point,^[^ ^35^, ^56^ ^]^ ii) thermal de‐pinning of domain walls from defects^[^ ^57^ ^]^ and/or iii) cross‐domain pinning effects potentially occurring during the re‐entrant relaxor transition, as discussed in Ref.^[^ ^29^ ^]^ With further increase of the temperature through the MT region, the δ_3_ is less affected and slightly shifted toward –180° (see yellow arrow). Finally, when entering the HT stage, an abrupt turn of the δ_3_ phasor is observed toward the middle of the green quadrant. At 302 °C, the phasor evolves toward δ_3_ = –130°, thus showing qualitatively the same characteristics as that of PMN–27PT (see Figure 4c). The results reveal the transitional nature of the third harmonic response evolving with temperature across distinct stages toward a reversible anhysteretic, divergent‐like component found previously in monoclinic PMN–xPT ceramics with strong relaxor behavior.^[^ ^10^ ^]^

At this point, it should be mentioned that the soft‐like response in BFO‐BTO in the HT region is different from that observed in hard (normal) ferroelectrics, where the reported superlinear behavior and third‐harmonic phase angle reflect strong defect‐mediated domain‐wall pinning effects.^[^ ^34^, ^58^, ^59^, ^60^ ^]^ Obviously, superliner behavior and divergent‐like hysteresis‐loop deformation, as summarized in Figure 4, cannot be straightforwardly assigned to the piezoelectric behavior of relaxor origins without proper supporting evidence, such as permittivity dispersion, domain‐switching behavior, and domain configuration.

Another point of discussion is the possible influence of the high‐driving electric fields, especially when approaching the coercive field (E_c_),^[^ ^60^, ^61^, ^62^ ^]^ on the observed superlinear field‐dependent $d_{33}^{\prime }$ (Figure 4d), large irreversible hysteresis (Figure 4e) and divergent‐like piezoelectric response (Figure 4f) of 0.67BFO in the HT region. To elucidate this point, we performed additional Rayleigh analysis to compare the temperature‐dependent piezoelectric coefficients ($d_{33}^{\prime }$, $d_{33}^{init}$ and α*) of 0.8BFO, 0.7BFO and 0.67BFO where $d_{33}^{\prime }$ and α* were calculated for fixed driving‐to‐coercive field ratio (E_0_/E_c_) of 0.2. The results are shown in Figure S11 (Supporting Information). At the same proximity to E_c_, the analyzed coefficients of 0.67BFO up to 300 °C are consistently higher than those of 0.8BFO and 0.7BFO and the pronounced increase in the irreversible coefficient (α*) is still observed in the HT region. These findings exclude the possible key role of the driving field proximity to E_c_ in the nonlinear responses and support the idea of the compositionally induced effect related to the relaxor behavior and thus domain structure, as presented in the following section.

### 2.4 Temperature‐Dependent Domain Structure at the Micro‐ and Nano‐Scale

**Figure 5:**
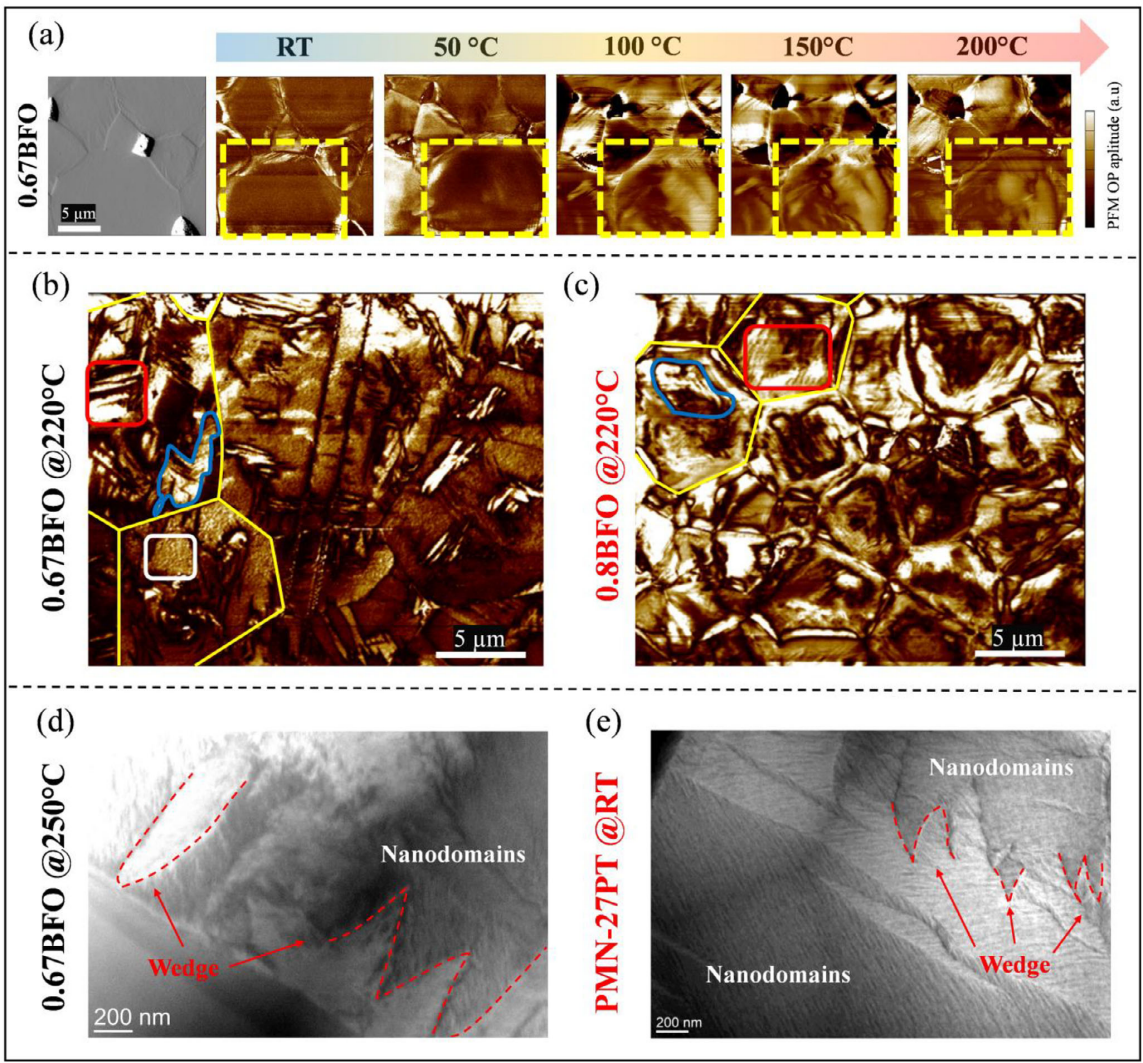
a) Atomic‐force microscopy topographical height image and piezo‐response force microscopy (PFM) out of plane (OP) images of 0.67BFO sample at different temperatures. The topographical image at room temperature is presented as a reference. The yellow rectangle highlights a region where the nucleation of macrodomains is observed. b) PFM OP image of 0.67BFO at 220 °C. The yellow lines indicate grain boundaries, while the red and white rectangles show representative regions of wedge‐like and nanocluster domains, respectively. An example of irregular domains is outlined in blue. c) PFM OP amplitude of the 0.8BFO sample for comparison. d) In situ transmission electron microscopy (TEM) image of the 0.67BFO sample at 250 °C. e) Room temperature TEM image of PMN–27PT shown for comparison. In both TEM images, the wedge‐like domains (dashed red) embedding the striation‐like nanodomains are highlighted.

To further elucidate the temperature dependence of the piezoelectric coefficient, we conducted a comparative analysis of the sample of interest (0.67BFO) and two reference samples (0.8BFO and PMN–27PT) using piezoresponse force microscopy (PFM) and transmission electron microscopy (TEM) at selected temperatures. We start with the temperature‐dependent evolution of domain configuration in the 0.67BFO sample measured using PFM, which is shown in **Figure** **5**a. At room temperature, the sample predominantly consists of regions inside grains with very weak PFM signal; however, in the vicinity of grain boundaries, the presence of ferroelectric domains can be observed, similar to those reported in a recent TEM study on Mn‐doped 0.75BFO.^[^ ^63^ ^]^ A closer look (data shown in Figure S12, Supporting Information) revealed the presence of nanodomains in the grain interior typical for relaxors. At 50 °C, no significant differences occur; however, at 100 °C, the domain configuration exhibits marked changes. PFM analysis at this temperature revealed the presence of well‐defined domains distributed homogeneously across the grain, whose concentration appears to increase with further increase of the temperature (see 150 and 200 °C). Considering that the macrodomains grow from nanodomain regions, we can interpret the domain evolution as a transition between ergodic relaxor and ferroelectric phases. This behavior, which is not common in normal ferroelectrics, is characteristic of re‐entrant relaxor‐type systems.^[^ ^29^, ^49^ ^]^ It should be noted that this domain evolution with temperature is fully consistent with the VF‐type relaxation observed in the LT region (see Figure 2c) and with several independent studies confirming the re‐entrant relaxor behavior in BFO–BTO.^[^ ^29^, ^49^, ^50^ ^]^ Note that the temperature of 100 °C, where the presence of macrodomains is first observed, is well consistent with the temperature at which the frequency‐dispersive permittivity exhibits an anomaly in the LT region (see Figure 2c), enhancing the link between the re‐entrant relaxor phenomenon and the temperature‐dependent domain evolution. The re‐entrant model, in principle, foresees an evolution with increasing temperature of the type relaxor → ferroelectric → relaxor; however, in reality, it could be much more complex as the coexistence of both states, in different proportions depending on the temperature, can be observed.^[^ ^64^ ^]^ A clearer example is provided in Figure 5b, where the domain configuration in the 0.67BFO sample heated at 220 °C presents multiple types of domains. Based on the morphological appearance, it is possible to clearly distinguish several types of domains: i) wedge‐like (see red rectangle) with an average size of 206 nm (see details about the statistical size analysis of domains in Figure S13, Supporting Information), ii) irregular domains (indicated by the blue line, which is purposely drawn to highlight the irregular shape of these domains), both containing iii) internal striation‐ or laminate‐type domains and nanocluster domains (a representative area is highlighted by a white rectangle) with an average size of 94 nm (Figure S13, Supporting Information). Importantly, the smallest nano features were not observed in the 0.8BFO sample heated to the same temperature (Figure 5c), meaning that they are either absent or present in a significantly smaller concentration. This BFO‐richer sample appears to exhibit a domain configuration similar to that of unmodified BFO, thus primarily consisting of wedge domains (with an average size of 132 nm; Figure S13, Supporting Information) and irregular domains;^[^ ^52^ ^]^ our analysis is also consistent with earlier reports on the PFM imaging of BFO–xBTO ceramic series.^[^ ^35^ ^]^ The domain structure of 0.8BFO ceramics with mostly macrodomains is consistent with the very weak relaxor feature of these samples (see Δ*T_m_* in Figure 1d).

Through TEM analysis of 0.67BFO performed at 250 °C (Figure 5d), it is possible to observe that the nanodomains are also arranged within the wedge‐like domains in a hierarchical‐type configuration, exactly as observed at room‐temperature in PMN–27PT (see, for comparison, Figure 5e). The nanodomains appear to adopt a configuration characterized by striation‐like patterns, whose characteristic appears to be maintained even at higher temperatures (see for example Figure S14, Supporting Information) at 375 °C, which is the temperature above which deploying sets is^[^ ^29^ ^]^). This structural arrangement is more clearly discernible through TEM imaging, offering valuable insights into the nanoscale organization of the material. In the case of PMN–27PT and similar monoclinic PMN–xPT compositions,^[^ ^10^ ^]^ we previously found that such domain configuration, originating from the relaxor disorder, is the key to the particular type of cascade‐like domain‐wall motion coupled to reversible (divergent‐like) dynamics, both reflected in the macroscopic response of PMN–27PT at room temperature and 0.67BFO in the HT region, as shown in Figure 4. The local cascade‐like DW switching is thus presumed to be responsible for the large nonlinearity and hysteresis, which is well supported by ex situ TEM and in situ XRD studies on PMN–PT.^[^ ^33^, ^65^ ^]^ On the other hand, the divergent‐like behavior originates from reversible DW dynamics, again consistent with previous in situ TEM observations.^[^ ^66^ ^]^ The results suggest a similar mechanism operating in lead‐free BFO–BTO at high temperatures (HT region) and in compositions with sufficient relaxor character. It should be noted that in BFO–BTO this particular dynamic response of relaxor origins may be additionally modified by the M‐W‐like redistribution of internal fields inside the ceramics, resulting in, e.g., large internal fields concentrated closer to grain boundaries in the HT region. This may lead to new and emerging phenomena, worthy of additional investigations.

Before concluding, we have to comment on possible influences on the large high‐temperature piezoelectric response of 0.67BFO other than those related to the relaxor behavior of the system. First, a possible transition from the oxygen‐octahedra‐tilted *R3c* phase to the non‐tilted *R3m* phase with increasing temperature and the associated depinning effects, as suggested by Xie et al., cannot be ruled out a priori.^[^ ^29^ ^]^ We emphasize, however, that i) a high‐temperature structural study on homogeneous ceramic samples would be necessary, considering that Ref.^[^ ^29^ ^]^ reports on a significant amount of core–shell‐like inhomogeneities and ii) previous systematic studies on tilted (*R3c*) and non‐tilted (*R3m*) PZT revealed that the pinning effects possibly arising from octahedra tilting is insignificant compared to the much stronger pinning effects arising from oxygen‐vacancies‐related defect complexes.^[^ ^67^ ^]^ Second, a large super‐coercive bipolar strain response was recently reported in BFO–BTO‐based ceramics, leading to an equivalent large‐signal $d_{33}^{*}$ coefficient of ≈1200 pm V^−1^ measured at 80 kV cm^−1^ (this field is an order of magnitude higher than used in our case).^[^ ^68^ ^]^ The fingerprint of the response is the highly asymmetric bipolar strain‐field hysteresis, consistently observed in other lead‐free piezoceramics, including (K,Na)NbO_3_‐based^[^ ^69^, ^70^ ^]^ and (Bi,Na)TiO_3_‐based ceramics.^[^ ^71^, ^72^, ^73^ ^]^ Recent discoveries suggest sample bending as the origin of the asymmetric strain‐field loops.^[^ ^74^, ^75^, ^76^ ^]^ The large strains due to bending should occur under specific conditions related to the clamping of the samples during measurements and in samples of low thicknesses, typically <0.5 mm,^[^ ^77^ ^]^ which is fairly lower than the thicknesses used during our high‐temperature piezoelectric measurements (≈2 mm).

## 3 Summary and Conclusion

In this study, we provide a systematic analysis of the piezoelectric response of the BFO–BTO system using a wide compositional range (from 0 to 50 mol% BTO), temperature range (up to 325 °C) and by varying the driving‐field conditions (amplitude and frequency). We demonstrate an exceptional converse piezoelectric coefficient of 1200 pm V^−1^ achieved at temperatures exceeding 300 °C. Three distinct temperature regions were identified in relation to the piezoelectric and dielectric response, contributing to the overall high‐temperature behavior. Specifically, we have determined that the first region (LT; ≈25 –125 °C) is associated with a re‐entrant relaxor transition, the second region (MT; ≈125–200 °C) exhibits characteristics of an Arrhenius‐type thermally activated conductivity process of M‐W origin, while the third region (HT; > 200 °C) is linked to the interface dynamics in hierarchically structured nanodomains. In relation to the HT contribution, we discovered that the nonlinear piezoelectric harmonic response of 0.67BFO at elevated temperatures (> 200 °C) resembles that previously found in a series of monoclinic PMN–xPT ceramic compositions (20 ≤ x ≤ 30) at room temperature. In particular, nonlinear reversible and irreversible responses were identified in the HT region in BFO‐BTO, the first characterized by a divergent‐like piezoelectric hysteresis‐loop deformation, while the second coupling a nonlinear response with a large non‐Rayleigh‐like hysteresis. These two dynamic contributions have been previously related to the particular reversible and irreversible (cascade‐like) domain‐wall displacements in disordered lead‐based relaxor ferroelectrics, indicating a similar mechanism operating in lead‐free BFO‐BTO at elevated temperatures (> 200 °C). This conclusion is supported by the common domain structure observed in the two material systems characterized by hierarchical arrangements of nanodomains.

We think that the results open new venues in optimizing the piezoelectricity of BFO–BTO and similar lead‐free piezoceramics. For example, a possible coupling between the MT and HT contributions in BFO–BTO may be envisioned where the effect of redistributed local electric fields in ceramics arising from the M‐W effects (e.g., large internal fields concentrated at grain boundaries) may be used to further boost the dynamics of the highly mobile domain walls in relaxors. This could be particularly relevant considering that, in general, domain type and density are largely affected by discontinuous strain/polarization states at grain boundaries.^[^ ^78^ ^]^ The experimental observation of the growth of domains from grain‐boundary regions during the LT transition opens a new possibility related to the coupling between the effect of locally redistributed internal fields at grain boundaries (MT) and the re‐entrant relaxor behavior (LT). In this view, it could be thus interesting to apply the interface‐engineering approaches that are commonly used in conventional dielectric ceramics for capacitor applications ^[^ ^79^ ^]^ to more complicated piezoelectric compositions with relaxor character. Finally, we also believe that the findings of this study provide valuable insights into the complex electromechanical behavior of BFO–BTO systems by revealing the key elements of the large piezoelectricity, i.e., the relaxor disorder (HT), local variation in the electrical conductivity (MT) and re‐entrant relaxor behavior (LT).

## 4 Experimental Section

The ceramic compositions (1‐x)BiFeO_3_–xBaTiO_3_ (x = 0.2, 0.3, 0.33, 0.36, 0.4, 0.5) abbreviated as 0.8BFO, 0.7BFO, 0.67BFO, 0.64BFO, 0.6BFO and 0.5BFO, were synthesized via reactive sintering method, utilizing a mechanochemically activated mixture of Bi_2_O_3_ (Thermo Scientific, 99.999%), Fe_2_O_3_ (Thermo Scientific, 99.995%), BaCO_3_ (Alfa Aesar, 99.8%), and TiO_2_ (Sigma–Aldrich, Rutile polymorph > 99.98%). To reduce the electrical conductivity and presence of secondary phases,^[^ ^20^ ^]^ the ceramics were doped using 0.1 wt. % of MnO_2_ (99.9%, Alfa Aesar), which was added to the mechanochemically activated powder for all six compositions. For further details about the processing method, consult Refs.^[^ ^20^, ^21^ ^]^ The activated and dried powders were pressed into 8 mm diameters pellets using a uniaxial press at 150 MPa and reactive sintered at different temperatures according to the composition, i.e, at 1000 °C for 12h (0.8BFO), 1040 °C for 6 h (0.7BFO, 0.67BFO and 0.64BFO), 1050 °C for 6h (0.6BFO) and 1060 °C for 6h (0.5BFO) using 5 °C min^−1^ of heating and cooling rate.

The crystal structure of the samples was analyzed using X‐ray diffraction (XRD) with a PANalytical X'Pert PRO high‐resolution diffractometer (CuKα1 radiation) equipped with a 100‐channel X'Celerator detector (PANalytical, Almelo, Netherlands). Diffraction patterns were collected within a range of 10° to 90° with a step size of 0.016° and an integration time of 100 s per step. Structure determinations were conducted using the MAUD program (Multiple Analysis Using Diffraction) employing the Rietveld refinement method.^[^ ^80^ ^]^ The diffraction patterns were refined using the *R3c* structural model for 0.8BFO and 0.7BFO compositions, while the *Pm‐3m* model was employed for the 0.67BFO, 0.64BFO, 0.6BFO, and 0.5BFO compositions. To facilitate a comparison between the R3c and Pm‐3m structures, the rhombohedral phase, typically indexed according to the hexagonal unit cell, can be transformed into an equivalent pseudo‐cubic unit cell using the following equations:^[^ ^81^, ^82^ ^]^

(1) $$a_{pc}=\sqrt[{3}]{\frac{\sqrt{3}a_{h}^{2}c_{h}}{12}}$$

(2) $$sin\frac{\alpha_{r}}{2}=\frac{3}{2\sqrt{3+{\left(\frac{c_{h}}{a_{h}}\right)}^{2}}}$$

(3) $$\alpha_{pc}=90-\frac{\sqrt{3}}{2}\left(60-\alpha_{r}\right)$$

In the above equation *a_h_* and *c_h_* are the hexagonal cell parameters, *a_pc_* is the calculated pseudocubic cell parameter, α_*r*_ is the inter‐axial angle for the hexagonal setting of *R3c* and α_*pc*_ is the inter‐axial angle angle for the rhombohedral phase in the pseudocubic setting. Figure 1b illustrates the results derived from the calculations using Equations (1), (2), and (3) for the *R3c* space group.

Synchrotron radiation powder diffraction data have been collected at the ID15A beamline of the European Synchrotron Radiation Facility (ESRF), using a wavelength of λ = 0.193725 Å, which corresponds to E = 64 keV. The sample was put in a kapton© capillary (diameter = 1 mm), and measured at room temperature. Diffracted photons were detected using Dectris Pilatus3×2M utilizing a CdTe sensor. The detector mask was created with the program FIT2D calibration and azimuthal integration was all performed using the program pyFAI.

The microstructure of the sintered pellets was analyzed with a scanning electron microscope (SEM, Verios G4 HP, Thermo Fisher Scientific) using an in‐lens detector for topographical images and a retractable solid‐state detector for back‐scattered electrons. Prior to examination, the samples were ground and finely polished following conventional metallographic procedures and thermally etched at 900 °C for 15 min. Grain size distribution was assessed by analyzing microstructural images using Image Tool software (version 3.0, United States). The average grain size was determined by the line interception method (ASTM Standard E112‐13). The density of the ceramics was determined using pycnometry (Micromeritics AccuPyc II 1340, United States), while the relative densities were calculated based on the theoretical density of BFO and BTO using the mixing rule based on Vegard's law. For further details about the microstructures of the samples, see Figure S15 (Supporting Information).

Piezo‐force microscopy (PFM) and conductive atomic force microscopy (c‐AFM) analyses were performed using an atomic force microscope (Asylum Research, Molecular Force Probe 3D, Santa Barbara, California, USA). Heating experiments were performed using a commercial heater (Polymer Heater, Asylum Research, Santa Barbara, California, USA) in combination with a high‐voltage PFM holder. Ti/Pt‐coated silicon tips on aluminium‐coated silicon cantilevers (OMCL‐AC240TM‐R3, Olympus, Japan) with a radius of 15 nm were used. PFM out‐of‐plane (OP) amplitude images were acquired using a PFM dual‐AC resonance tracking switching spectroscopy (DART‐SS) mode by applying an AC voltage with an amplitude of 5 V and a frequency of ≈350 kHz between the conductive AFM tip and the bottom electrode (Ted Pella Silver Paste, product number 16035, Redding, California, USA). c‐AFM experiments were performed by applying a DC voltage between the tip and a bottom electrode in ORCA mode (ORCA, Asylum Research, Molecular Force Probe 3D). The DC voltage used for c‐AFM imaging was 100 V.

A transmission electron microscope (JEM‐2100, Jeol Ltd., Tokyo, Japan) operated at 200 kV with a beryllium double‐tilt specimen holder (Gatan Inc.) was used to analyze the domain structure of the ceramics. In situ heating TEM experiments in the temperature range of 20–450 °C were carried out in order to monitor the domain structure changes and phase transition behavior. Images at elevated temperatures were acquired after 5 min holding time at a specific temperature.

For electrical and electromechanical measurements, the opposite surfaces of the samples were coated with magnetron‐sputtered Au electrodes (5 Pascal, Italy). The permittivity versus temperature curves were obtained using an automated temperature‐controlled furnace with an impedance analyzer (Hioki IM3536 LCR meter) for measurements of capacitance in parallel configuration (C_p_) and dielectric losses. Measurements were conducted at varying frequencies (1, 10, 100 kHz) from room temperature up to different maximum temperatures depending on the composition, with a heating and cooling rate of 2 °C min^−1^. The 0.8BFO samples were characterized using a high‐temperature experimental setup (max temperature ≈1000 °C). Measurements were conducted using a ProboStat system (NorECs AS) enclosed within an alumina tube. An S‐type thermocouple was used for temperature detection and platinum (Pt) electrodes were connected to the sample. Data acquisition and monitoring were performed using Omega software interfaced with a Keithley DMM6500 multimeter and an MFIA (Zurich Instruments) impedance analyzer. In preparation for the experiment, a thin layer of gold paste was applied to both primary surfaces of the specimen using screen printing methodology. Following the application, the sample underwent thermal treatment at 720 °C for a duration of 15 min to allow electrode sintering and ensure robust adhesion.

To perform the Vogel‐Fulcher analysis on 67BFO and 0.5BFO samples, the permittivity peak temperatures at distinct frequencies were extracted from the datasets using a wider frequency range scan (10 Hz to 100 kHz) for a total of 21 frequencies. The Vogel‐Fulcher parameters were obtained by fitting the experimental data according to the Vogel‐Fulcher equation:

(4) $$f=f_{0}\cdot e^{-\frac{E_{a}}{k\left(T_{m-}T_{f}\right)}}$$

where *T_m_* is the permittivity peak temperature at a given frequency, *E_a_* is the activation energy, *T_f_* is the Vogel‐Fulcher freezing temperature, *f_0_* is the relaxation frequency at infinite temperature, and *k* is the Boltzmann constant. Despite the strict validity of V‐F relation for canonical relaxors and the fact that several relaxation mechanisms can contribute to ɛ(T) maximum and thus *T_m_* ^[^ ^64^ ^]^, for simplicity and to cross‐compare permittivity dispersions in different temperature regions, Equation (4) and the parameters as originally defined by V‐F law were still used.

Polarization and strain versus electric field were measured with a TF2000 ferroelectric/piezoelectric test system (aixACCT, Germany) using a single sinusoidal waveform at 10 Hz of driving field frequency and applying 60 kV cm^−1^ for all compositions, except for 0.8BFO, which was measured at 120 kV cm^−1^.

Samples were poled in silicone oil at room temperature by applying 40–80 kV cm^−1^ of DC electric field for 20 min, depending on the exact composition, i.e, 40 kV cm^−1^ for 0.5BFO, 0.6BFO, 0.64 BFO and 0.67 BFO; 45 kV cm^−1^ for 0.7BFO and 80 kV cm^−1^ for 0.8BFO.

Piezoelectric d_33_ coefficients were measured using a standard Berlincourt piezometer (Take Control PM10, Birmingham, UK), working at 110 Hz and using a dynamic force of 0.25 N.

Nonlinear piezoelectric harmonic measurements as a function of temperature were performed using a homemade setup consisting of a fiber‐optic sensor for displacement measurements (MTI 2100 Fotonic Sensor, USA). For the electric driving of the samples, a voltage generator (SRS DS360, USA) and a voltage amplifier (TREK 609E‐6, USA) were used. Detection of the first and third harmonic signals of the piezoelectric strain was performed by two lock‐in amplifiers (SR830 DSP, USA), while the piezoelectric hysteresis was acquired using an oscilloscope (Keysight InfiniiVision 1000 X‐Series, USA). All measurements were performed at 10 Hz of driving field frequency. Additional details of the analysis were reported in Refs.^[^ ^10^, ^34^ ^]^

Temperature‐dependent harmonic measurements were performed in two different experimental configurations, i.e., dynamic and isothermal. Dynamic measurements were performed by heating the sample with a constant heating rate of 2 °C min^−1^ and by simultaneous acquisition of the first harmonic component of the piezoelectric and dielectric response. The applied sinusoidal electric‐field amplitude and frequency were 1 kV cm^−1^ and 90 Hz, respectively. The dielectric harmonic response was measured using the shunt‐resistor method and lock‐in technique as explained in detail in Ref.^[^ ^10^ ^]^ Note that the distance between the fiber‐topic probe and the reflecting surface, consisting of a golden cantilever mechanically pressing the piezoelectric samples (like used in Ref.^[^ ^83^ ^]^), changes during dynamic measurements due to thermal expansion effects. This leads to a change in the sensitivity factor. To account for this temperature‐dependent sensitivity factor, the experimental data were corrected by determining the sensitivity factor for different probe‐to‐target distances using a micrometer screw. Unlike dynamic measurements, isothermal piezoelectric harmonic analysis was performed by heating the sample at a defined temperature, where, after thermal equilibration of the sample, the first and third piezoelectric harmonic responses were collected with two lock‐in amplifiers as a function of increasing electric‐field amplitude using the same setup as already described.

## Conflict of Interest

The authors declare no conflict of interest.

## Supporting information



Supporting Information

## Data Availability

The data that support the findings of this study are available from the corresponding author upon reasonable request.
