# Supplementary material for: Large High‐Temperature Piezoelectric Response of Lead‐free BiFeO3–BaTiO3 Originating from Relaxor Disorder
Source: Small. 2025 Jun 9;21(31):2502379. doi: 10.1002/smll.202502379 (PMC12332824; doi:10.1002/smll.202502379)
Supplement: Supplementary file 1 — Supporting Information [file SMLL-21-2502379-s001.docx]

**Supplementary Materials**

**Large high-temperature piezoelectric response of lead-free BiFeO_3_–BaTiO_3_ originating from relaxor disorder**

Antonio Iacomini, Janina Roknić, Issa Sidibe, Marco Scavini, Mojca Otoničar, Hana Uršič, Tadej Rojac

**Supplementary Material 1:** Rietveld refinement of X-ray diffraction (XRD) data

**
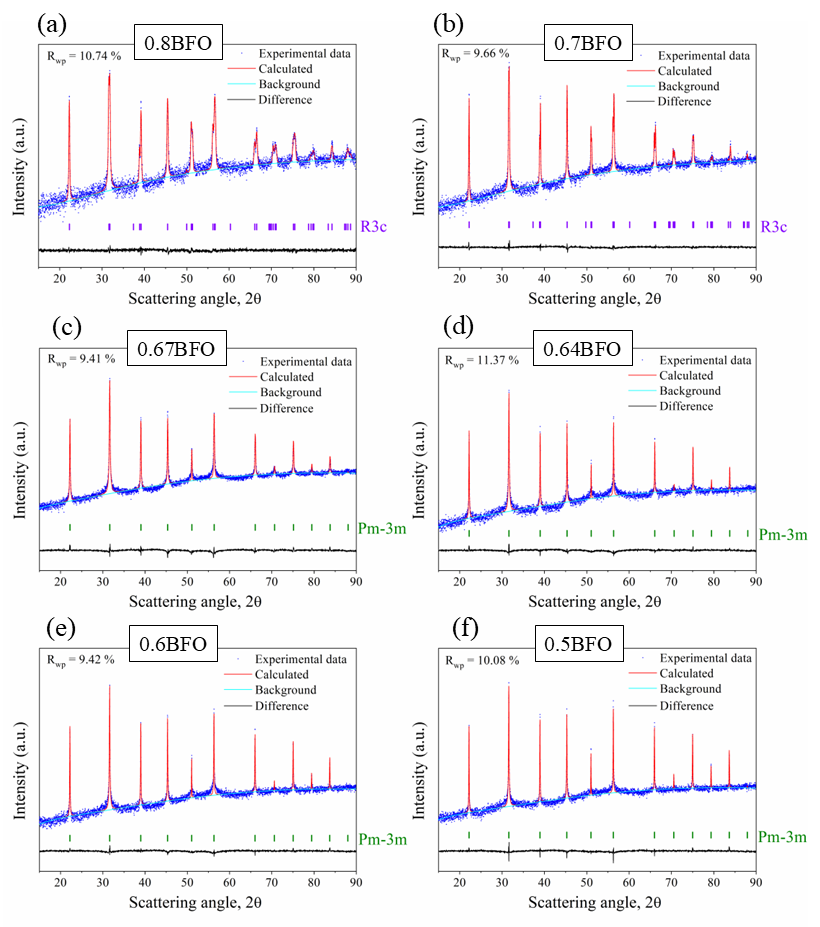
**

**Figure S1.** XRD analysis and Rietveld refinement of (a) 0.8BFO, (b) 0.7BFO, (c) 0.67BFO, (d) 0.64BFO, (e) 0.6BFO and (f) 0.5BFO. Blue dots are experimental data points, red curves are the calculated diffraction curves according to the model chosen for each sample, i.e, *R3c* for 0.8BFO and 0.7BFO, and *Pm-3m* for 0.67BFO, 0.64BFO, 0.6BFO and 0.5BFO. The bar sequence marks the expected peak position from the values of lattice parameters and space group of the crystallographic phases. The black line at the bottom represents the residuals, i.e., the difference between the square root of experimental and calculated intensities point-by-point. The cyan color line represents the calculated background. The weighted profile residual (R_wp_) calculated for each refinement is reported as an inset. Intensities of the diffraction pattern are reported in logarithmic scale to better highlight any secondary phases and peaks at high angles. No additional secondary peaks can be clearly identified in all the samples.

The diagnostic (111) peak for the BTO-richer compositions, namely 0.64BFO, 0.6BFO, and 0.5BFO, highlighted in **Figure 1a** of our manuscript, exhibits a high degree of symmetry without any evident peak splitting (within the resolution limits), thus confirming the cubic-like symmetry. Regarding the 0.67BFO sample (please refer to manuscript **Figure 1a**), we can draw similar conclusions based on the same single-peak characteristic; therefore, the average structure of 0.67BFO is also most likely pseudocubic. This is further corroborated by our synchrotron data and Rietveld analysis (see **Figure S4**) and supported by several independent studies in the literature ^[1,2]^.

The question now arises whether the 0.8BFO and 0.7BFO samples, which exhibit (111)-type peak splitting indicative of rhombohedral structures (as seen in manuscript **Figure 1a**), present a certain percentage of cubic phase that may increase with the increasing BTO component. We have carried out additional Rietveld analyses for the 0.8BFO and 0.7BFO samples, in which the cubic phase was added in the refinement. The fitted results were then compared with those using the single-phase analysis shown in **Figure S1**. Additionally, for the purpose of proper comparison, we also conducted a fitting procedure using the single-cubic phase, so that all possibilities are considered and evaluated. The results of these fittings are shown in **Figures S2** and **S3** for the 0.8BFO and 0.7BFO samples, respectively.

As shown in **Figures S2(a1,a2)** and **S3(a1,a2)**, the single-cubic model fails to describe the experimental data, as demonstrated by the high figure of merit (R_wp_>14%) obtained in both cases. The single-rhombohedral model (**Figures S2(b1,b2)** and **S3(b1,b2)**) exhibits much better fit (R_wp_ ~10% in both cases), while the combination of rhombohedral and cubic phases (**Figures S2(c1,c2)** and **S3(c1-c2)**) shows a slight improvement in the figure of merit (R_wp_ ~10% and R_wp_ ~9% for 0.8BFO and 0.7BFO, respectively). While we acknowledge the slight improvement in the figure of merit, which is less than 1% in both cases, we conclude that this enhancement is not substantial enough to warrant the inclusion of the cubic phase into the fit. Furthermore, the peaks associated with the cubic phase completely overlap with those of the rhombohedral phase, making accurate quantification particularly challenging and, most likely, impossible with the given resolution quality of the data. Given these considerations, we conclude that the simpler model without the cubic phase provides a more reliable and justifiable representation of our data.

**
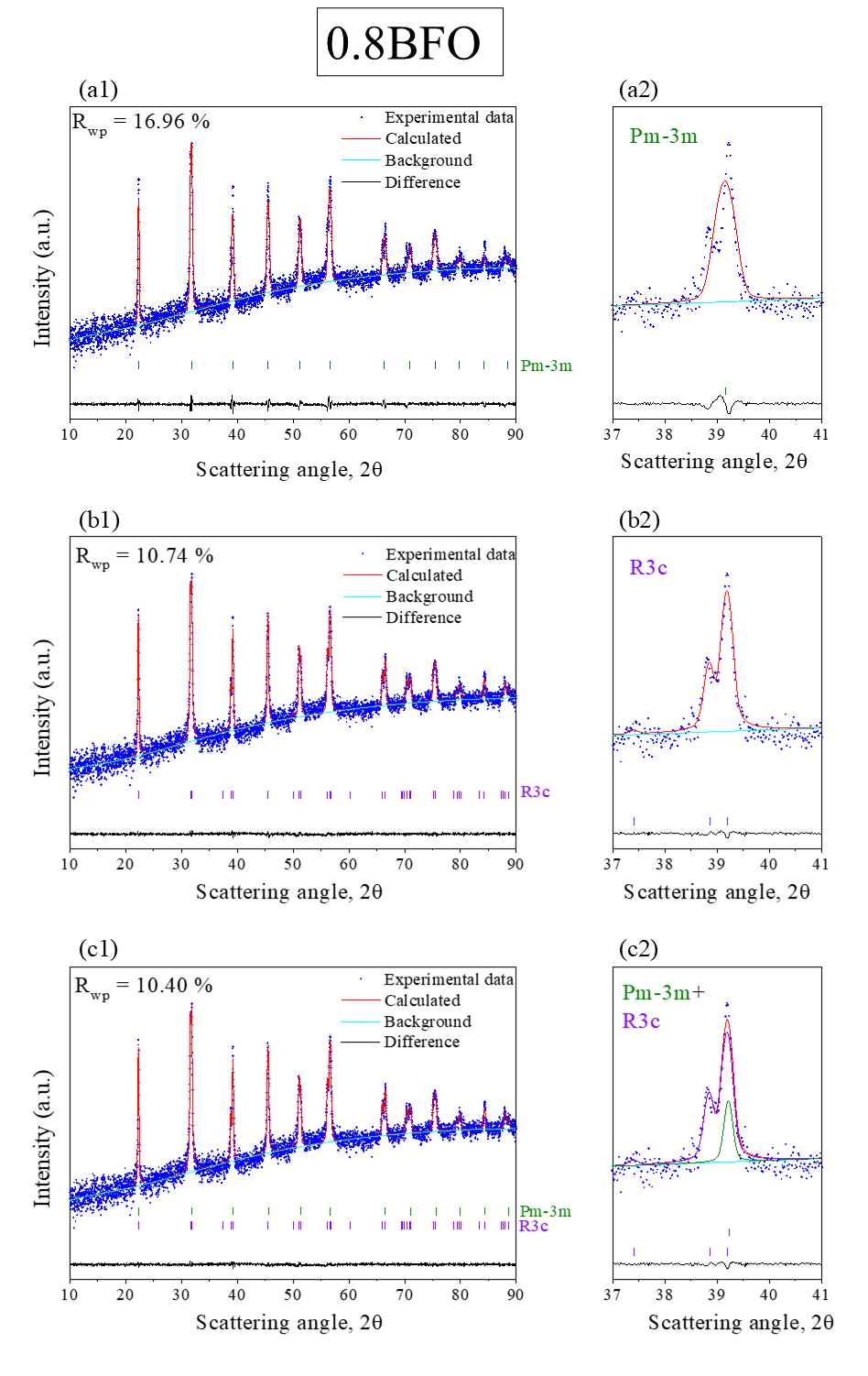
**

**Figure S2**. Comparison of Rietveld refinements of the 0.8BFO sample using three distinct structural models: (a1) single cubic phase (Pm-3m), (b1) single rhombohedral phase (R3c), and (c1) coexistence of rhombohedral and cubic phases (R3c+Pm-3m). Panels a2-c2 displays magnified views of the diagnostic (111)_pc_ diffraction peak using the three different models. Intensities are expressed on a log scale.

**
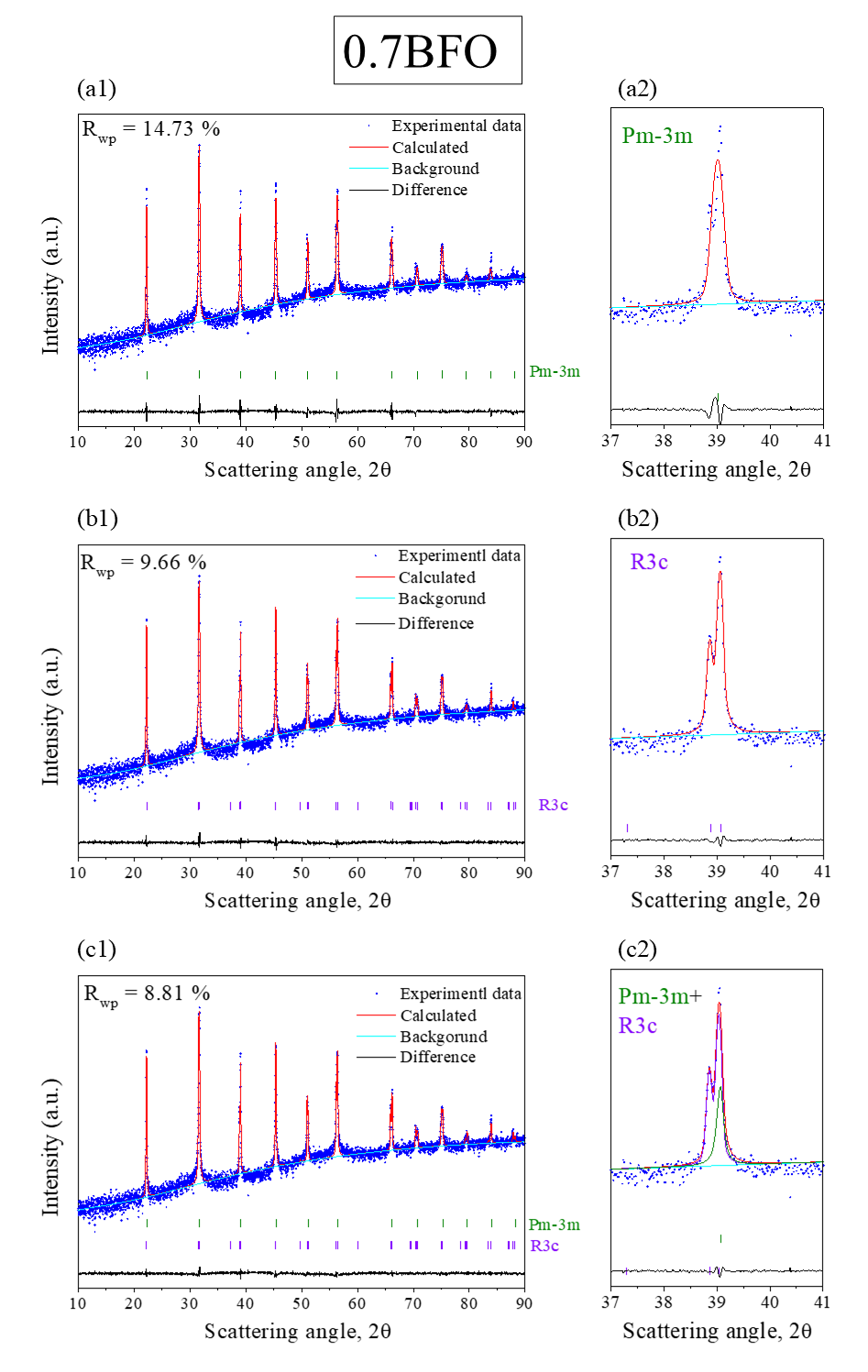
**

**Figure S3**. Comparison of Rietveld refinements of the 0.7BFO sample using three distinct structural models: (a1) single cubic phase (Pm-3m), (b1) single rhombohedral phase (R3c), and (c1) coexistence of rhombohedral and cubic phases (R3c+Pm-3m). Panels a2-c2 displays magnified views of the diagnostic (111)_pc_ diffraction peak using the three different models. Intensities are expressed on a log scale.

**Supplementary Material 2:** Synchrotron X-ray diffraction analysis of 0.67BFO sample


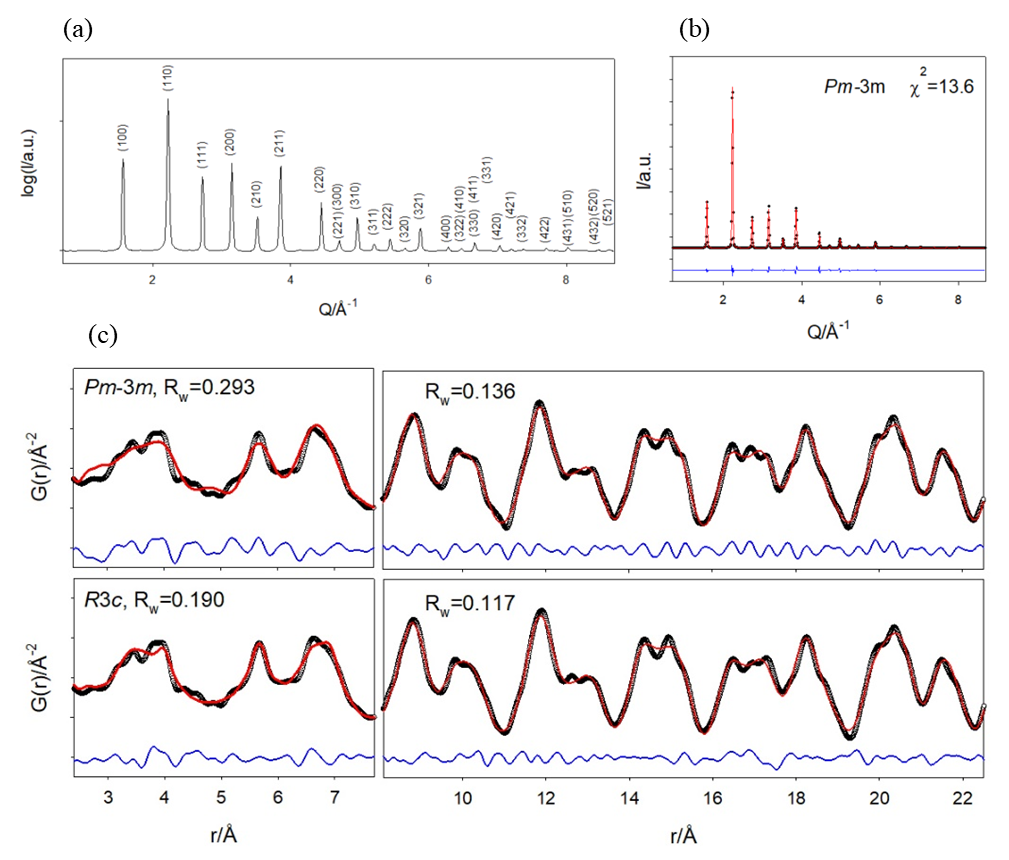


**Figure S4.** (a) Experimental diffraction pattern (black curve) collected on the 0.67BFO sample in logarithmic scale after background subtraction. The numbers in brackets are the Miller indices of Bragg peaks using the cubic *Pm-3m* structural model of the primitive undistorted perovskite. (b) Experimental data (black symbols) and calculated pattern using the *Pm-3m* model (red curve), together with the difference between the experimental and calculated patterns (blue curve). (c) Experimental G(r) functions of 0.67BFO in the 2.4-7.7 Å and 8-22.5 Å ranges (black circles). The calculated G(r) in the two ranges using either the *Pm-3m* (top) or the *R3c* (bottom) models are shown as red curves, while the curves difference is shown in blue. The fit residuals R_w_ are also indicated on the respective plots.

In the case of symmetry lower than cubic, peaks splitting and/or new (superstructure) Bragg peaks should appear, which was not observed in the experimental pattern shown in **Figure S4a**, despite the good signal-to-noise ratio. As a consequence, the Rietveld refinement was applied to the experimental data using the ideal cubic *Pm-3m* model; the refinement is shown in **Figure S4(b)**, while the calculated parameters are reported in **Table S1**. The refined value of the atomic main square displacement U value for the Bi/Ba cations (U(Bi/Ba) in **Table S1**) is as large as ≈0.07 Å^2^, a very high value for a room temperature measurement. For example, for BiFeO_3_ at room temperature, a value of U(Bi)=0.008 Å^2^ was reported, which is an order of magnitude lower than determined here for 0.67BFO ^[3]^. The increase of U values in solid solutions relative to that in the parent compound at fixed temperature is a fingerprint of structural disorder ^[4]^.

We suppose that U(Bi/Ba) includes a non-negligible contribution from disorder, ascribable to the different electronic configurations of the two cations shearing the perovskite A site: while Ba^2+^ is a close-shell cation, the lone pair of Bi^3+^ should lead to the shift of the Bi^3+^ cation position away from the center of its cubocthaedral oxygen cage due to the presence of the lone-pair electrons ^[3]^.

**Table S1.** Parameters calculated according to the Rietveld refinement using the *Pm-3m* model. *a*, *b*, *c* are the cell parameters; α, β, γ are the cell angles; *U* is the atomic main square displacement; *R_w_* and χ^2^ are the goodness-of-fit parameters used to evaluate the quality of the fit.

| Space group | *Pm-3m* |
| --- | --- |
| a=b=c (Å) | 3.9942(3) |
| α=β=γ (°) | ≡90 |
| U(Bi/Ba) (Å^2^) | 0.073(1) |
| U(Fe/Ti) (Å^2^) | 0.030(1) |
| U(O) (Å^2^) | 0.055(3) |
| R_w_ (/) | 0.056 |
| χ^2^ (/) | 13.6 |

To reveal and map the structural disorder, we carried out the analysis of the same data in the real space, using the so-called pair distribution function (PDF) analysis. Since the G(r) function consists of both contributions of Bragg peaks and diffuse scattering, it also reveals short-range correlations and symmetry breaking. In the first step, the experimental G(r) was fitted in the 2.4-7.7 Å range of the interatomic distances r, to map the local disorder using the *Pm-3m* and *R3c* models. In fact, we cannot exclude the presence of local symmetry breaking consistent with the space group of the *R3c* model, which merges into a more symmetric structure at the average scale (see, e.g., the recent multiscale structural study on Pb(Mg_1/3_Nb_2/3_)O_3_ ^[5]^). To be noted is that the *R3c* model is tested here, considering that it is the ground state of BFO ^[6]^. The left side panels of **Figure S4(c)** show the experimental data (empty circles) together with the fits (red curve) and the residuals (blue curve) for the *Pm-3m* (top) and the *R3c* (bottom) models.

As observed in **Figure S4c**, the *R3c* model supplies less residuals than the *Pm-3m* one. However, both fail in accurately fitting the experimental G(r) function, especially in the shortest distances range (1.7-4.0 Å), which corresponds roughly to one perovskite cell. This reflects the complex local structure of the system and suggests that each A cation tends to reconstruct locally its “ideal” environment. The right-hand side panels of **Figure S4(c)** show the fits of the experimental G(r) using the same models in an “intermediate” range of interatomic distances, that is 8-22.5 Å. Also in this range, the $R3c$ model supplies a better fit than Pm-3m, although the difference is smaller compared to that in the 7.7 Å distance range (**Figure S4c**, left). The better fit of the G(r) data using the rhombohedral model is consistent with previous local-structure studies on BFO-BTO ^[7]^ and suggests that polar displacements exist with a coherence length of at least 2 nm, which is typical for relaxors; in fact, a similar observation has been recently reported for the canonical relaxor PMN ^[5]^. As a final comment, whatever the model considered, the atomic mean square displacement of the A cations U(Bi/Ba) is always as large as ≈0.06 Å^2^, in agreement with Rietveld results (≈0.07 Å^2^; see **Table S1**, confirming that the coexistence of Bi^3+^ and Ba^2+^ ions on the A site introduces large structural disorder).

**Supplementary Material 3:** Vogel-Fulcher (V-F) analysis of 0.67BFO and 0.5BFO

**
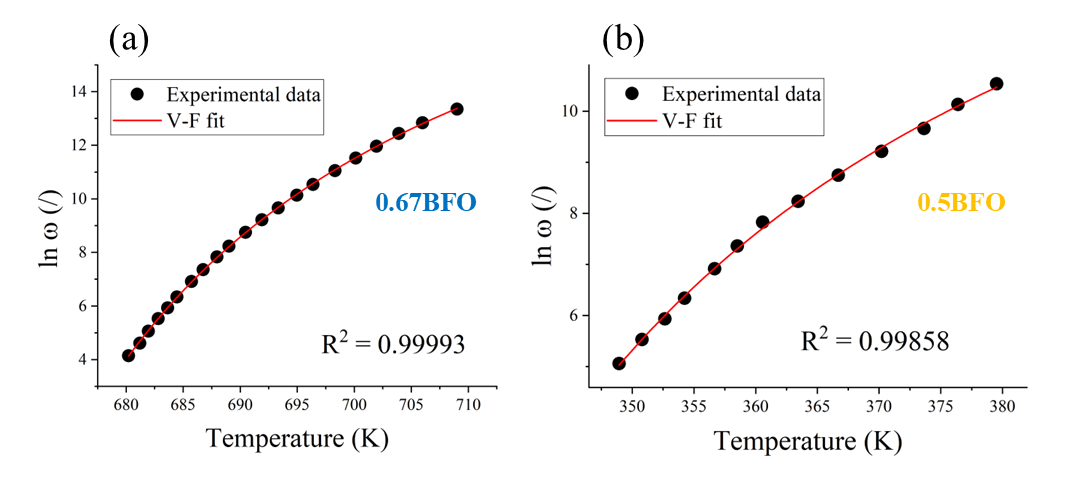
**

**Figure S5.** Vogel-Fulcher plots for (a) 0.67BFO and (b) 0.5BFO showing the natural logarithm of angular frequency ω plotted against the temperature (T_m_) measured at a given frequency. The full lines represent the fitted functions (**Equation 4** in the experimental section) while black dots are experimental data. The goodness of fit *R²* is given on the respective plots.

**Table S2**. Fitted parameters determined from Vogel-Fulcher analysis of the dielectric response of 0.67BFO and 0.5BFO compositions. The pre-exponential factors f_0_ are not shown due to unreliability related to the analysis performed over a limited frequency range, particularly in the case of the 0.5BFO sample.

| Sample | E_a_ (eV) | T_f_ (°C) |
| --- | --- | --- |
| **0.67BFO** | 0.065 | 370 |
| **0.5BFO** | 0.067 | 23 |

**Supplementary Material 4:** Evolution of the coercive field (E_c_) as a function of the temperature

**
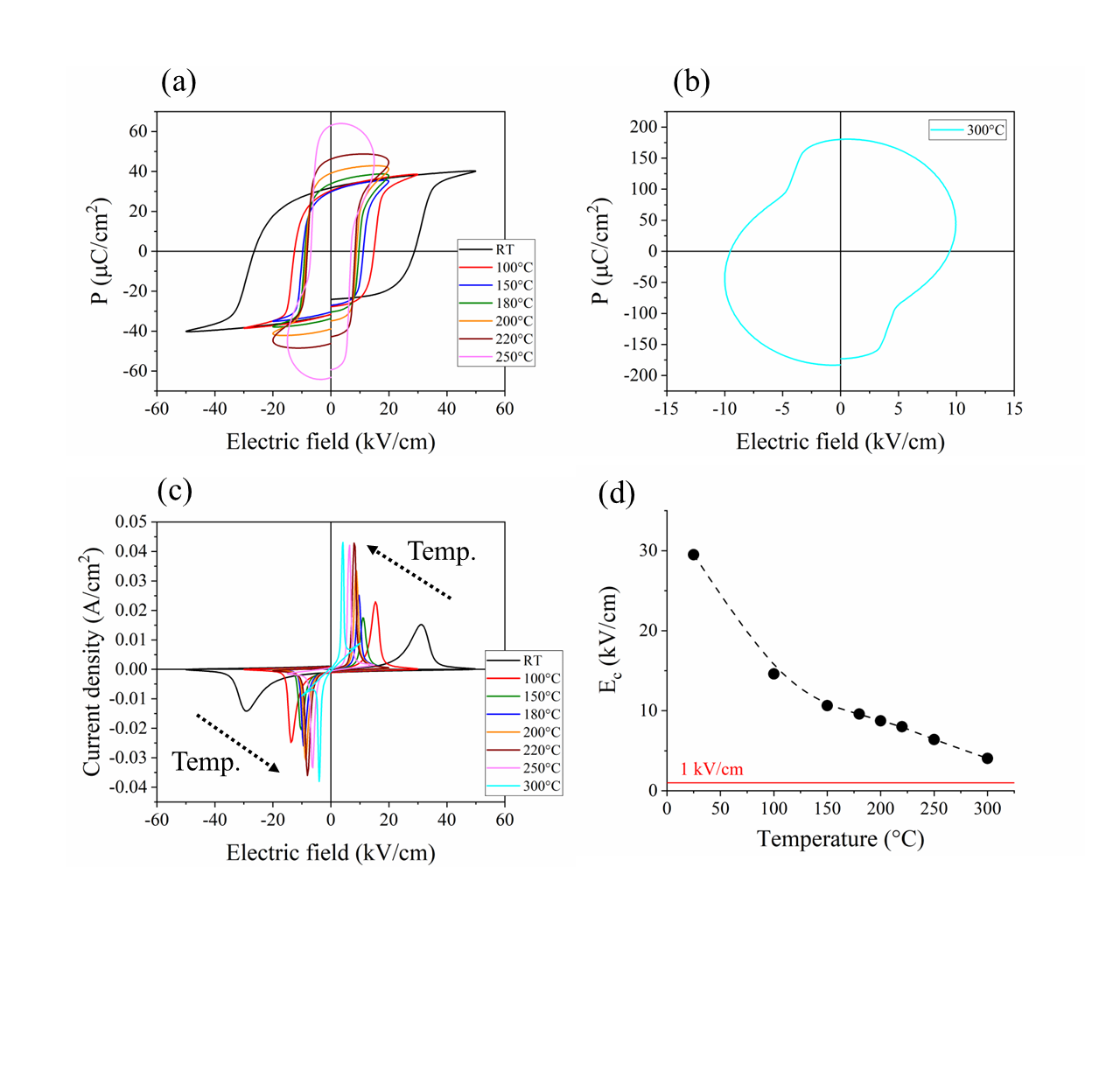
**

**Figure S6**. (a,b) Polarization (P) and (c) current-density loops of 0.67BFO measured at different temperatures. The hysteresis loop measured at 300°C, depicted in panel (b), is presented separately for clarity of the hysteresis visualization. All measurements were conducted at 10 Hz. (d) Evolution of the extracted coercive field (E_c_) as a function of temperature. Each data point was obtained by averaging the two points from the peak current (see panel (c)). The red line denotes the maximum electric-field amplitude (1 kV/cm) used during the temperature-dependent piezoelectric characterization, presented in the main paper (Figure 2). The comparison confirms that the piezoelectric measurements were conducted using subcoercive driving fields.

**Supplementary Material 5:** Frequency, electric-field amplitude and temperature dependence of the converse piezoelectric response of 0.67BFO


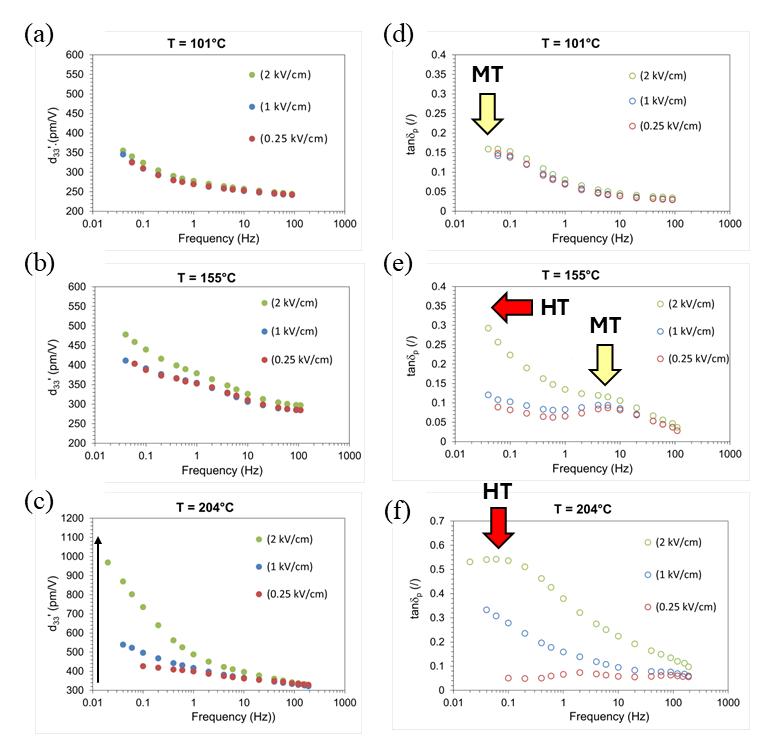


**Figure S7**. (a,b,c) Converse piezoelectric $d_{33}^{'}$ coefficient and (d,e,f) tangent of the piezoelectric phase angle, ${tan\delta}_{p},$of 0.67BFO sample as a function of frequency for different driving electric field amplitudes, measured at three different temperatures: (a,c) 101°C, (b,e) 155°C and (c,f) 204°C. The data show a series of relaxations identified by a peak in ${tan\delta}_{p}$ accompanied by an increase in $d_{33}^{'}$ with decreasing frequency. Yellow and red arrows in panels d,e,f indicate the ${tan\delta}_{p}$peaks associated with MT and HT contributions, respectively (see also descriptions of these contributions in the main text of the paper associated with **Figure 2b**). It is important to note that the peak in ${tan\delta}_{p}$associated with the MT contribution shifts toward higher frequencies with increasing temperature due to thermal activation (see arrow MT in panels d and e) but remains practically independent of the applied electric field. In contrast, the HT peak demonstrates not only thermal activation (shift of peak toward higher frequencies with increasing temperature) but also electric field activation (see arrow HT in panels e and f). The latter is best observed by the large $d_{33}^{'}$ coefficient of 1000 pm/V observed at 204°C and 0.02 Hz (panel c), which is emerging from the response with increasing electric-field amplitude (from 0.25 to 2 kV/cm; see arrow in panel c).

**Supplementary Material 6:** Temperature dependence of the converse piezoelectric and dielectric response of 0.67BFO sample


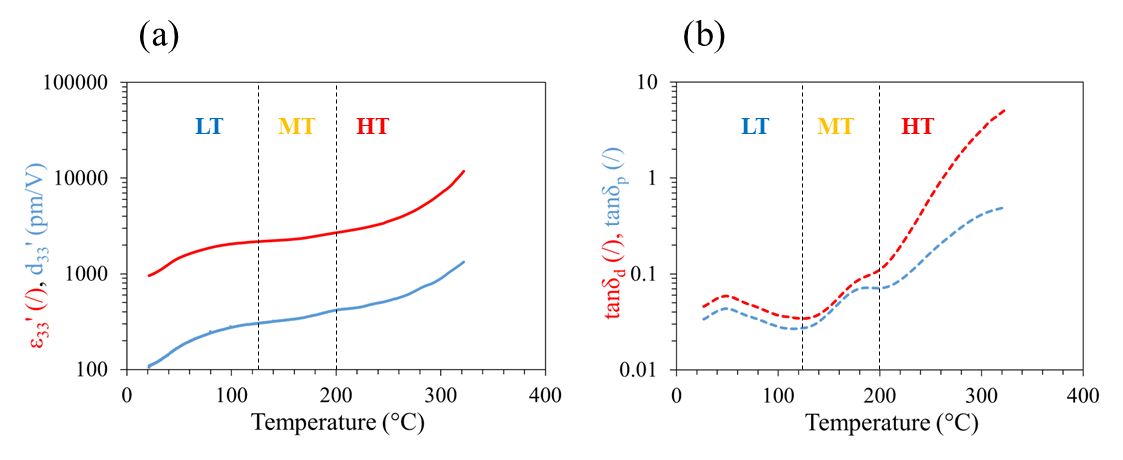


**Figure S8**. (a) Temperature dependence of piezoelectric $d_{33}^{'}$ coefficient (blue curve) and dielectric permittivity $\varepsilon_{33}^{'}$ (red curve) of poled 0.67BFO sample. (b) Corresponding tangent of the piezoelectric (${tan\delta}_{p}$; blue-dashed curve) and dielectric phase angle (${tan\delta}_{d}$; red-dashed curve). The data were obtained by simultaneous measurements of the converse piezoelectric and dielectric response using 90 Hz of frequency and 1 kV/cm of driving-field amplitude (for further details about the measurements, see experimental section). The individual stages, i.e., LT, MT and HT, are noted on the graphs (see main paper, particularly Figure 2b, for details). The data reveal a consistency between the piezoelectric and dielectric response over the three stages (i.e., the temperature trend of the piezoelectric and dielectric curves is qualitatively the same).

**Supplementary Material 7:** Vogel-Fulcher (V-F) analysis of 0.67BFO

**
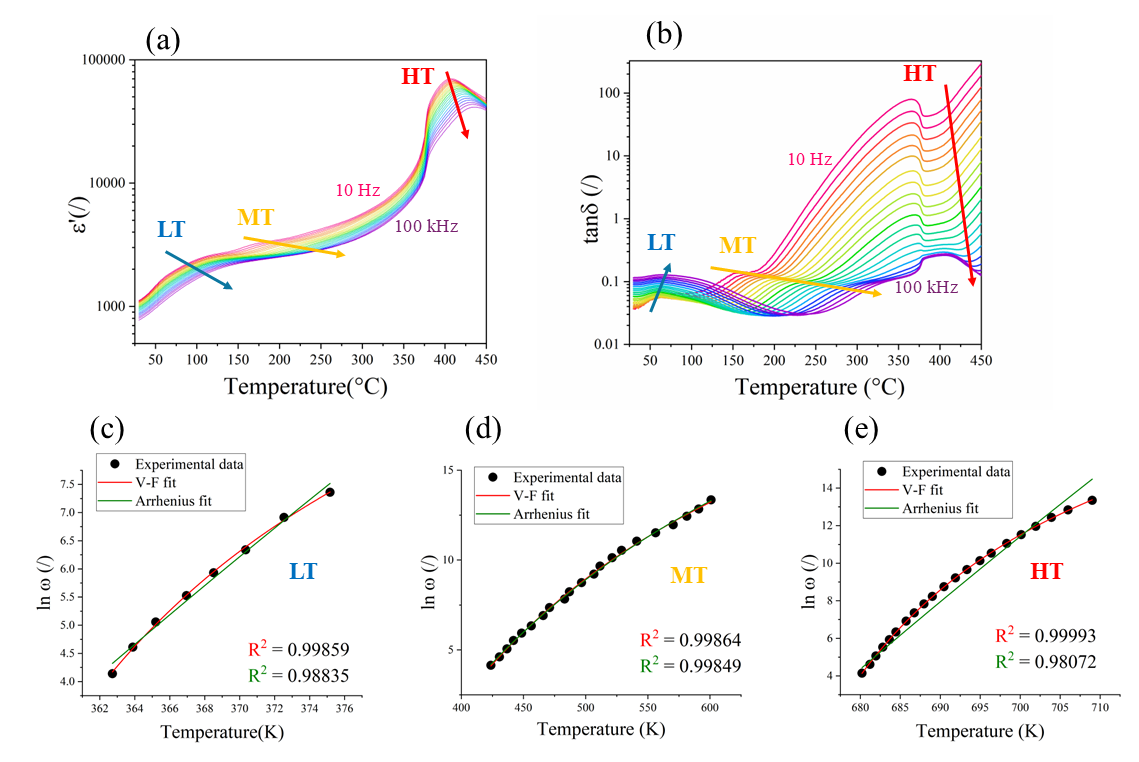
**

**Figure S9.** (a) Real part of the dielectric permittivity $(\varepsilon^{'}$) and (b) dielectric loss tangent (tanδ) versus temperature of 0.67BFO during heating cycle measured at different driving-field frequencies (10 Hz-100 kHz). Note the logarithmic vertical scales. The different stages identified (LT, MT, HT) are indicated within the panels. (c), (d) and (e) panels show Vogel-Fulcher and Arrhenius fitting for LT, MT and HT stages, respectively. The black points represent the experimental data, while the red line shows the fit using the Vogel-Fulcher (V-F) model, and the green line displays the fit obtained using the Arrhenius model. The goodness of fit, represented by the coefficient of determination (*R²*), is displayed within each panel for the respective fit.

The fitting analysis of the dielectric data of 0.67BFO (panels a, b) revealed that in the case of LT and HT the best fit was obtained with the V-F model (see panels c, e), which suggests a relaxor-like freezing phenomenon, while in the case of MT, both models converge to similar *R^2^* results (see panel d). The V-F fit of MT leads to a freezing point (T_f_) of -207°C, which is close to 0 K (-273°C), effectively corresponding to the Arrhenius law. In addition, the activation energy *E_a_* after fitting the data in the MT stage using the V-F model shows an order of magnitude higher value (0.85 eV) than those obtained by fitting the VF relaxations, i.e., LT (0.025 eV) and HT (0.065 eV) (all the fitted parameters are reported in Table 1 of the main paper). Both these factors work in favour of MT thermal activation, obeying more closely the Arrhenius law, rather than the V-F law.

**Supplementary Material 8:** Observation of Maxwell-Wagner (M-W) effect and electrical conductivity activation process in 0.8BFO

**
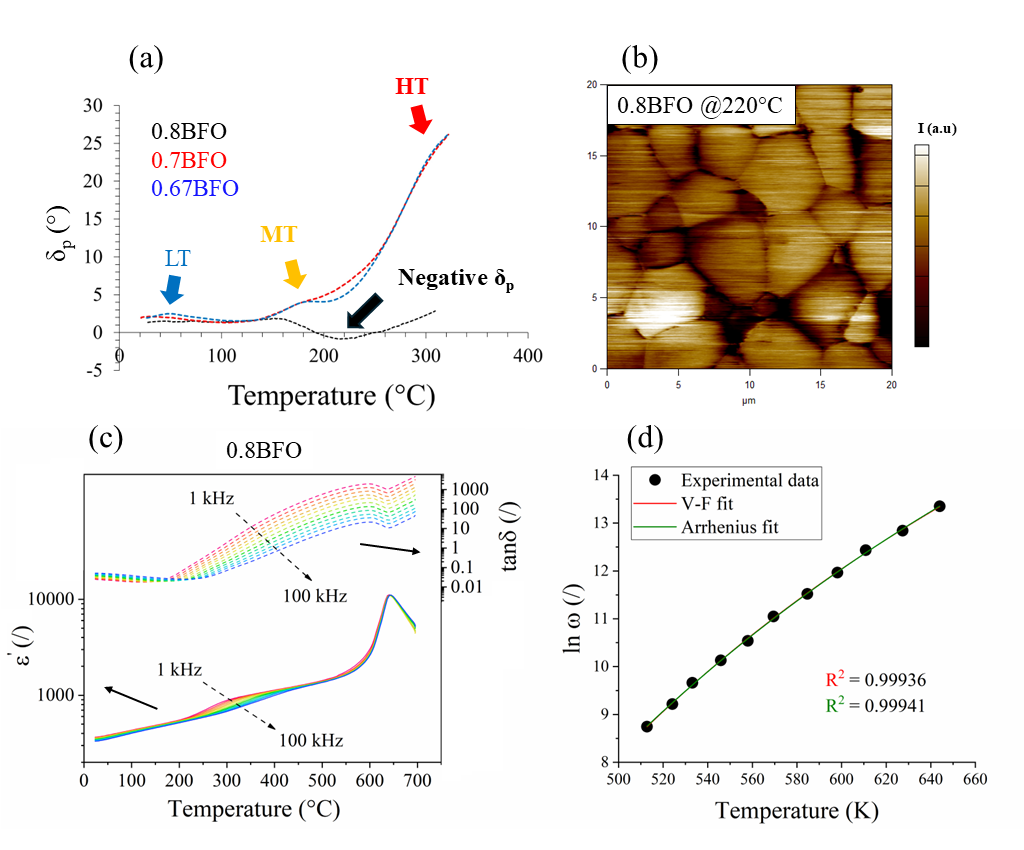
**

**Figure S10.** (a) Temperature evolution of the piezoelectric phase angle *δ_p_* of 0.8BFO, 0.7BFO and 0.67BFO samples. Distinct phase-angle peaks are associated with each stage (LT, MT, and HT) and are represented by different coloured arrows in the graph. Note the negative phase angle (indicated with black arrow) identified in the 0.8BFO sample at around 220°C. (b) Conductive atomic-force microscopy (c-AFM) image of 0.8BFO sample at 220°C clearly showing that the grain boundary regions exhibit a lower local electrical conductivity than the grain interiors (see dark regions corresponding to lower local electric-current signal; I – electric current). Both the negative piezoelectric phase angle and the presence of insulating grain boundaries, identified at the same temperature of 220°C, are consistent with the M-W effect (further details and supporting references are presented in the main paper). (c) Real part of the dielectric permittivity $(\varepsilon^{'}$) and dielectric loss tangent (tanδ) versus temperature of 0.8BFO sample during heating cycle measured at different driving-field frequencies (1 – 100 kHz). Note the vertical logarithmic scales. (d) Vogel-Fulcher and Arrhenius fitting of the dispersion of 0.8BFO ceramics in the temperature range between 200 and 450°C. This dispersion is highlighted by dashed arrows in panel (c).

**Table S3.** Fitted parameters calculated from Vogel-Fulcher and Arrhenius analyses of the dielectric responses of 0.67BFO and 0.8BFO samples. The data for 0.67BFO correspond to the MT stage and are the same as those given in **Table 1**; they are shown here again for the sake of direct comparison with the fitted parameters obtained from the analysis of the 0.8BFO sample. The results suggest that the dispersions observed in 0.8BFO and 0.67BFO samples (MT stages) are of the same nature, i.e., they show Arrhenius-type thermal activation with Ea close to 1 eV. Since piezoelectric and c-AFM analysis of 0.8BFO sample (**Figure S11a,b**) show clear signatures of piezoelectric M-W effect, we infer a similar mechanism governing the MT stage of 0.67BFO ceramics.

| Sample | Model | E_a_ (eV) | T_f_ (°C) |
| --- | --- | --- | --- |
| **0.67BFO** | Arrhenius | 1.13 | / |
|  | Vogel-Fulcher | 0.85 | -207 |
| **0.8BFO** | Arrhenius | 1.00 | / |
|  | Vogel-Fulcher | 0.87 | -236 |

**Supplementary Material 9:** Rayleigh analysis

This section contains the details regarding Rayleigh-law (RL) properties and analysis that were used in the current study. For more explanations, we suggest that the reader consults Ref. ^[8]^

RL consists of a set of two equations. The first equation describes the linear relationship between the piezoelectric coefficient and electric-field amplitude:

$d^{'}\left( E_{0} \right)=d^{init}+\alpha\cdot E_{0}$ (S1)

where$d^{'}$, $d^{init}$, $\alpha$ and $E_{0}$ are the real-part of the piezoelectric coefficient, reversible coefficient, irreversible coefficient and electric-field amplitude, respectively. For any given $E_{0}$, $d^{init}$ represents the field-independent fraction of the total piezoelectric coefficient $d^{'}\left( E_{0} \right)$, while the field-dependent fraction is given by $\alpha\cdot E_{0}$. Note that in **Equation S1** RL is expressed for the converse piezoelectric response with the matrix notation (e.g., subscripts 33 for the longitudinal effect) omitted for clarity.

The second RL equation relates the strain response ($x$) as a function of the electric field ($E$),

describing the weak-field *x*–*E* piezoelectric hysteresis loop as:

$x\left( E \right)=(d^{init}+\alpha\cdot E_{0})\cdot E\pm\frac{\alpha}{2}(E^{2}-E_{0}^{2})$ (S2)

where + and – signs stand for decreasing and increasing field, respectively. Note that the second term, which describes hysteresis, contains the irreversible $\alpha$ coefficient, illustrating the characteristic relationship between hysteresis (second term of **Equation S2**) and nonlinearity (**Equation S1**). As a common practice, **Equation S2** is used to predict the RL hysteresis based on the $d^{init}$ and $\alpha$ coefficients determined by fitting the experimental $d^{'}\left( E_{0} \right)$ data using **Equation S1**. This principle was also used for the calculation of the so-called RL hysteresis shown in **Figure 4b,e** of the main paper.

In the case of ferroelectric materials, RL describes macroscopic weak-field hysteresis (**Equation S2**) assuming a microscopic mechanism consisting of reversible and irreversible displacements of domain walls (or similar interfaces) in a medium with randomly distributed pinning centers (usually considered as point defects) ^[9–12]^. Note that RL originally defines a linear relationship between the coefficient and field amplitude (see **Equation S1** where $\alpha$ is constant, i.e., field independent), which has been rationalized in terms of the “randomness” of the pinning potential ^[13]^. In most real cases, however, the piezoelectric coefficient tends to deviate from the ideal linear field relationship, particularly at high driving fields (see, e.g., Refs ^[9,13,14]^). Without going deeply into the complex underlying mechanisms responsible for these deviations, an effective and pragmatic approach to the macroscopic analysis is to use a modified RL equation of the type ^[9,13,15]^:

$d^{'}\left( E_{0} \right)=d^{init}+\alpha^{*}\cdot E_{0}$ (S3)

where

$\alpha^{*}\left( E_{0} \right)=\alpha_{1}+\alpha_{2}\cdot E_{0}+\alpha_{3}\cdot E_{0}^{2}+\ldots$ (S4)

In the above equations, $\alpha^{*}$ is defined as the field-dependent irreversible coefficient described by a polynomial function. Considering that the 0.67BFO composition in this study shows the piezoelectric $d_{33}^{'}$ coefficients mostly deviating from a perfectly linear relationships with $E_{0}$ (see **Figure 3a** of the main paper), we used the modified RL relations (**Equations S3** and **S4**) to extract the $d^{init}$ coefficient (**Figure 3c** of the main paper) and the $\alpha^{*}$ coefficient (plotted in **Figure 3c** of the main paper for different driving fields). A second-order polynomial function (**Equation S4**) was found to be sufficient to obtain good fits (R-Squared ≥ 0.999) to the experimental $d_{33}^{'} vs. E_{0}$ data shown in **Figure 3a** of the main paper.

The next analysis that we used in our study is the expansion of the RL law into Fourier series by assuming an alternating driving electric field in the form:

$E=E_{0}\cdot\sin\left( \omega t \right)$ (S5)

where $\omega$ is the driving electric-field (angular) frequency.

In this case, the Fourier series of **Equation S2** can be derived as ^[16]^:

$x\left( t \right)=\left( d^{init}+\alpha\cdot E_{0} \right)E_{0}\sin\left( \omega t \right)-\frac{4\alpha E_{0}^{2}}{3\pi}cos\left( \omega t \right)-\frac{4\alpha E_{0}^{2}}{3\pi}\left( \frac{1}{5}cos\left( 3\omega t \right)-\frac{1}{35}cos\left( 5\omega t \right)+\ldots\right)$ (S6)

To understand the individual elements of the Fourier expansion of RL (**Equation S6**), we must introduce the general expression for a nonlinear strain response in term of the Fourier series ^[16,17]^. By excluding the static term, the expression using two different forms is

$x\left( t \right)=\sum_{n=1,2,3\ldots} x_{0n}\sin\left( n\omega t+\delta_{n} \right)=\sum_{n=1,2,3\ldots} \left( x_{n}^{'}\sin\left( n\omega t \right)+x_{n}^{''}\cos\left( n\omega t \right) \right)$ (S7)

where

$x_{0n}=\sqrt{\left( x_{n}^{'} \right)^{2}+\left( x_{n}^{''} \right)^{2}}$ (S8)

$\tan\delta_{n}=\frac{x_{n}^{''}}{x_{n}^{'}}$, $x_{n}^{'}=x_{0n}\cdot cos\left( \delta_{n} \right)$, $x_{n}^{''}=x_{0n}\cdot sin\left( \delta_{n} \right)$ (S9)

$d^{'(n)}=\frac{x_{n}^{'}}{E_{0}}$, $d^{''(n)}=\frac{x_{n}^{''}}{E_{0}}$, (S10)

In the above equations *n* is the number of the harmonic, $x_{0n}$, $x_{n}^{'}$ and $x_{n}^{''}$ are the total, in-phase and out-of-phase strain amplitudes of the n^th^ harmonic, respectively, $\delta_{n}$ is the phase angle, and $d^{'(n)}$ and $d^{''(n)}$ are the real and imaginary coefficients of the n^th^ harmonic, respectively.

By using **Equation S7** as a reference and considering a sinusoidal input electric field (**Equation S5**), it becomes clear that the first (orange) term in the RL expansion in **Equation S6** represents the first harmonic in-phase strain (sine term), while the second (red) term represents the first harmonic out-of-phase strain (cosine term). Similarly, the terms noted in blue and green in equation S6 are higher harmonic contributions to the total strain, which are all odd (*n* = 3, 5, …) and out-of-phase (only cosine terms) with respect to the sinusoidal electric field (**Equation S5**). This means that the nonlinear terms (*n* = 3, 5, …) are all hysteretic (cosine), again illustrating the nonlinearity-hysteresis relationship of RL ^[16]^. By comparing **Equation S6** and **Equation S7**, several important properties of an ideal RL behavior with a perfectly random pinning-center distribution can be identified, which can be used as reference points in the evaluation of a particular experimental nonlinear response. The most important that was used in our analysis (**Figure 4c,f**) is the prediction of the third harmonic phase angle, $\delta_{3}$, of –90°.

Finally, in **Figure 3d** of the main paper, we analyse the fractional reversible (R) and irreversible (IR) contributions to the total $d_{33}^{'}\left( E_{0} \right)$ coefficient. The R contribution can be expressed as a percentage ratio between the R (field-independent) part of the total piezoelectric coefficient (equal to $d_{33}^{init}$; see **Equation S1**), and the total piezoelectric coefficient $d_{33}^{'}\left( E_{0} \right)$. Similarly, the IR contribution can be expressed as a percentage ratio between the IR (field-dependent) part of the total piezoelectric coefficient (equal to $d_{33}^{'}\left( E_{0} \right)-d_{33}^{init}=\alpha^{*}E_{0}$; see **Equation S1**) and total piezoelectric coefficient $d_{33}^{'}\left( E_{0} \right)$.

**Supplementary Material 10:** Piezoelectric response of 0.8BFO, 0.7BFO and 0.67BFO as a function of temperature at fixed driving-to-coercive field ratio

**
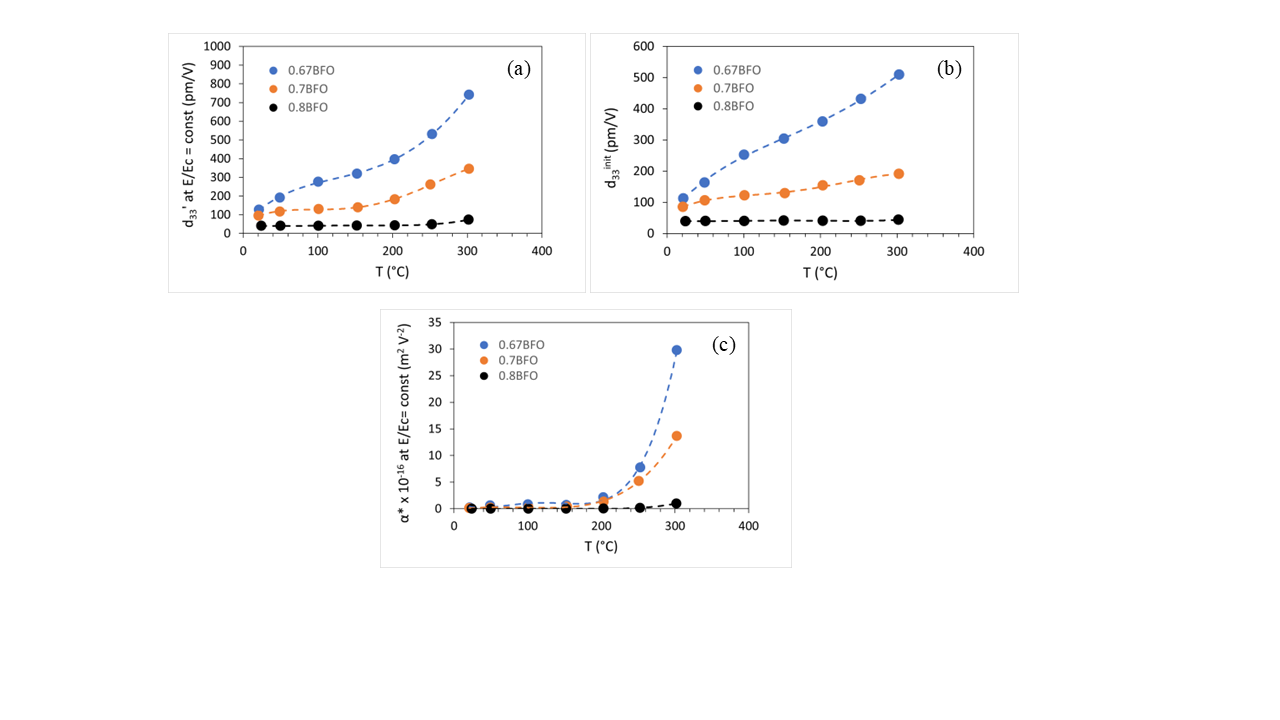
**

**Figure S11.** (a) Converse piezoelectric $d_{33}^{'}$ coefficient, (b) $d_{33}^{init}$ and (c) α* coefficients for 0.67BFO, 0.7BFO and 0.8BFO as a function of temperature. The $d_{33}^{'}$ and α* coefficients are plotted at fixed driving-to-coercive field ratio (E_0_/E_c_) ratio of 0.2. At fixed E_0_/E_c_ ratio, the three coefficients are consistently higher for 0.67BFO in the whole temperature range. These results exclude the possible dominant role of the driving field proximity to E_c_ in the large piezoelectric response of 0.67BFO and also in the large nonlinearity (α*) induced in the HT region above 200°C (see panel c). The temperature-dependent coercive field for 0.8BFO and 0.7BFO was determined using the same method as for the 0.67BFO (see supplementary material 4).

**Supplementary Material 11:** Piezo-response force microscopy **(**PFM) analysis of the domain structure of 0.67BFO sample at room temperature


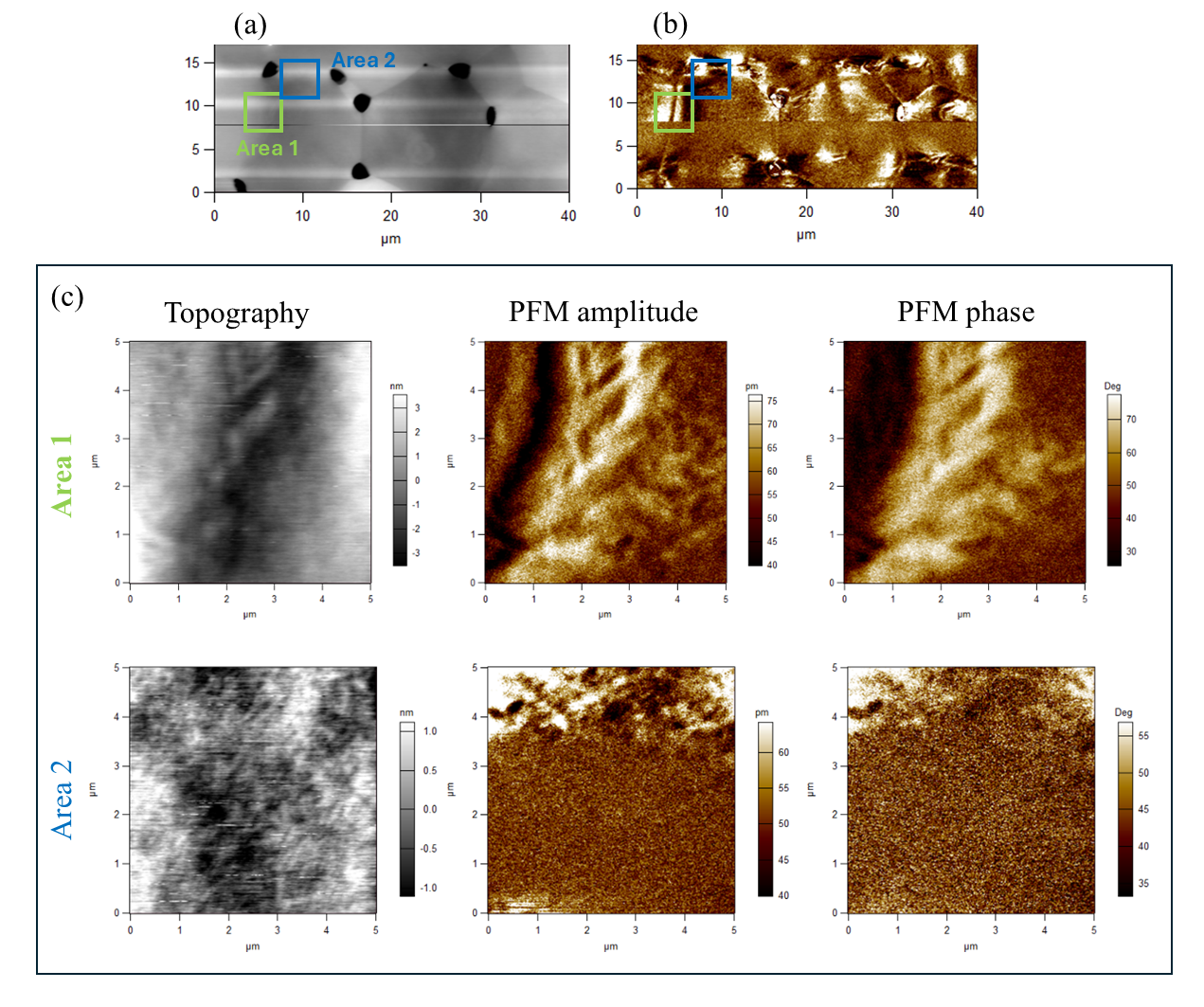


**Figure S12**. Piezoresponse Force Microscopy (PFM) analysis of 0.67BFO at room temperature. (a) Topography image. (b) PFM out-of-plane (OP) amplitude image. (c) Magnified views of two selected areas near grain boundaries, as indicated by green and blue rectangles in panels (a) and (b). The domain structure exhibits a clear presence of segregated macrodomains near the grain boundaries, while the grain core is characterized by nano-features typical of relaxor systems (see, e.g., Refs ^[18,19]^).

**Supplementary Material 12:** Domain size analysis using PFM imaging


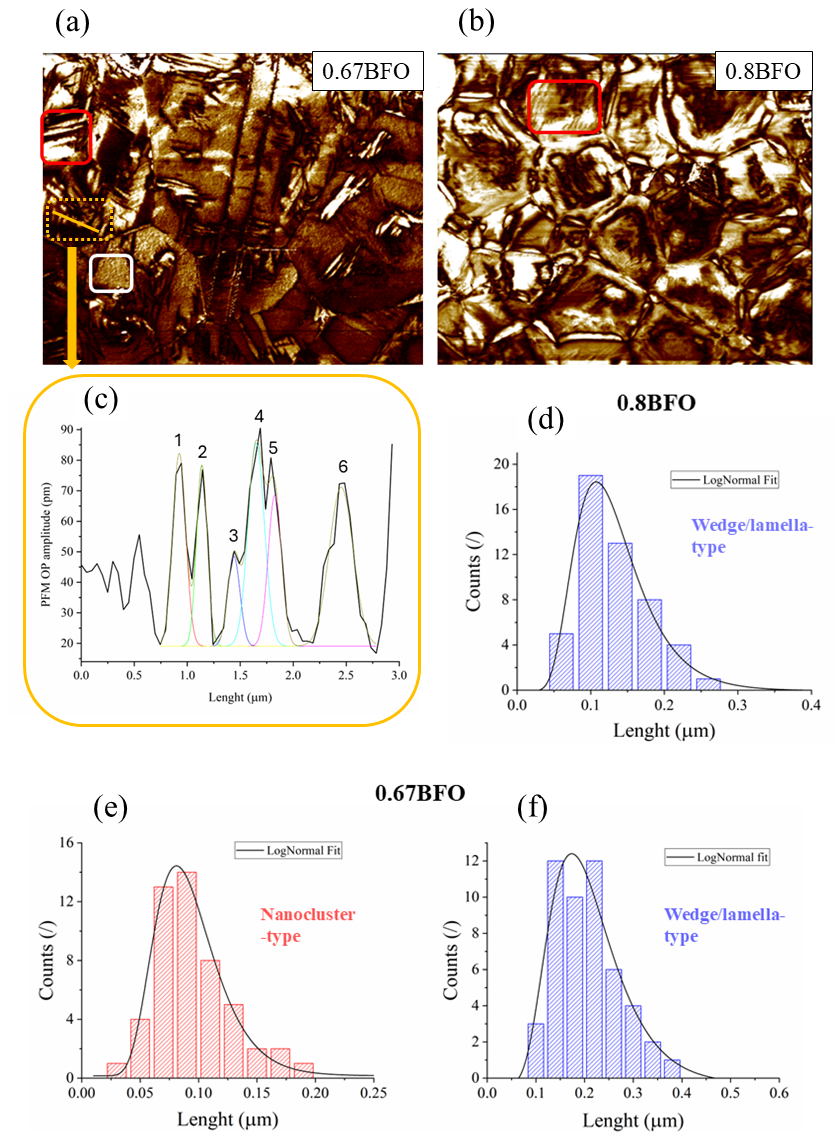


**Figure S13**. PFM-OP images at 220°C of (a) 0.67BFO and (b) 0.8BFO ceramics. Red squares in (a) and (b) highlight wedge/lamella-type domains, while the white square in (a) shows a representative zone of nanocluster-type domains. (c) Example of a line profile analysis showing the PFM-OP amplitude as a function of the length of the line, as marked in panel (a). Each peak corresponds to a single domain. (d-f) Histograms obtained from domain size analysis of 0.67BFO and 0.8BFO (at 220°C).

Analysis of in-situ PFM measurements at 220°C for the 0.67BFO and 0.8BFO samples revealed two distinct domain configurations, highlighted by red and white rectangles in Figure **S13a,b**. These configurations correspond to wedge/lamella and nanocluster-type domains, respectively. The 0.67BFO sample exhibits both domain types, while the 0.8BFO sample is predominantly characterized by wedge/lamella domains. To conduct a statistical analysis of domain sizes, we employed the following methodology. For each identified region, we drew multiple lines perpendicular to the domain orientation. We collected data from 50 peaks, with each peak representing a single domain. For illustrative purposes, we provide an example in **Figure S13c**. In this example, we analysed six peaks (numbered accordingly), each fitted with a Gaussian curve. Domain size was determined using the full width at half maximum (FWHM) of these curves. The results of our analysis are presented as histograms in **Figure S13d–f**. All three histograms display a log-normal distribution. The statistical analysis outcomes are summarized in **Table S4** below.

**Table S4.** Results of the statistical analysis of domain sizes of 0.67BFO and 0.8BFO samples at 220°C, showing the total number of analyzed domains (N_tot_) and the extracted mean and median sizes.

| **Sample/type of domain** | **N_tot_** | **Mean (µm)** | **Median (µm)** |
| --- | --- | --- | --- |
| 0.67BFO / nanocluster type | 50 | 0.09 | 0.09 |
| 0.67BFO / wedge/lamella type | 50 | 0.21 | 0.20 |
| 0.8BFO / wedge/lamella type | 50 | 0.13 | 0.13 |

**Supplementary Material 13:** In-situ TEM of 0.67BFO sample at 375°C





**Figure S14**. In-situ TEM of 0.67BFO at 375°C, showing the persistence of the hierarchical arrangement of domains with striation-like nanodomains inside larger wedge-like domains, like observed at 220°C (see **Figure 5d** in the main paper).

**Supplementary Material 14:** SEM characterisation of BFO–BTO series


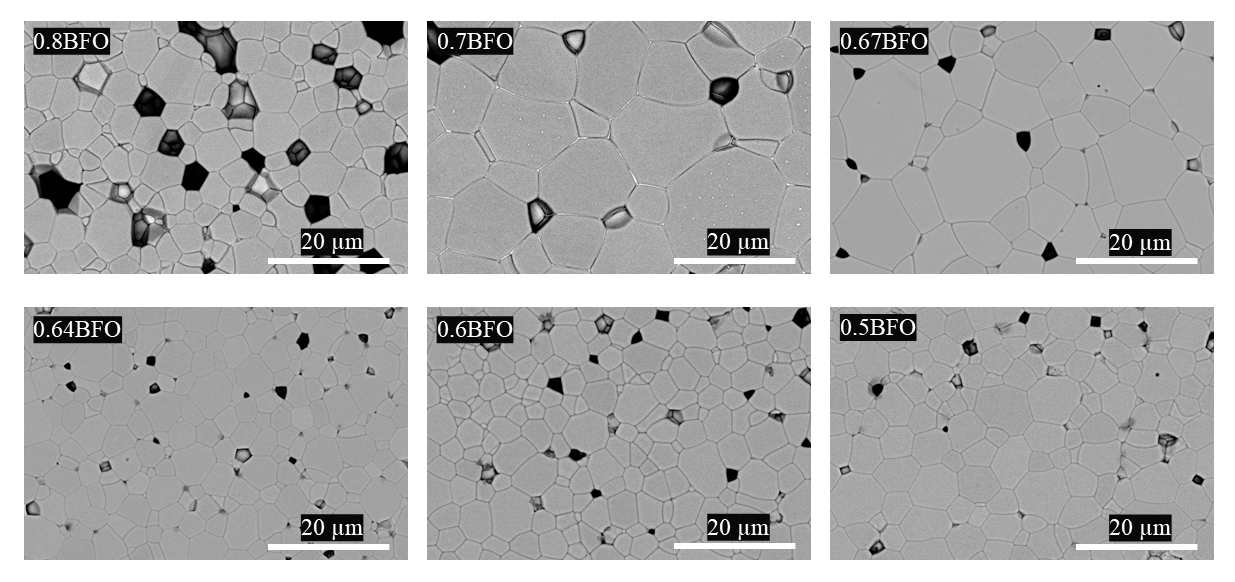


**Figure S15**. Backscattered Electron – Scanning Electron Microscopy (BSE-SEM) micrographs of thermally etched BFO-BTO ceramics.

**Table S5.** Relative density (ρ %) and average grain size (Avg. G.S) of BFO-BTO ceramics.

|  | 0.8BFO | 0.7BFO | 0.67BFO | 0.64BFO | 0.6BFO | 0.5BFO |
| --- | --- | --- | --- | --- | --- | --- |
| ρ relative (%) | 91 | 94 | 95 | 97 | 94 | 95 |
| Avg. G.S (µm) | 3.9 | 8.9 | 7.9 | 3.6 | 3.7 | 4.2 |

**References**

[1] Leontsev S. O., Eitel R. E., Dielectric and Piezoelectric Properties in Mn-Modified (1−x)BiFeO_3_–xBaTiO_3_ Ceramics. *Journal of the American Ceramic Society* **2009**, *92*, 2957.

[2] Ferrero G., Astafiev K., Ringgaard E., de Oliveira L. S., Sudireddy B. R., Haugen A. B., Žiberna K., Malič B., Rojac T., Piezoelectric Properties of Mechanochemically Processed 0.67BiFeO_3_-0.33BaTiO_3_ Ceramics. *Journal of the European Ceramic Society* **2023**, *43*, 350.

[3] Palewicz A., Przeniosło R., Sosnowska I., Hewat A. W., Atomic Displacements in BiFeO_3_ as a Function of Temperature: Neutron Diffraction Study. *Acta Cryst B* **2007**, *63*, 537.

[4] Scavini M., Coduri M., Allieta M., Masala P., Cappelli S., Oliva C., Brunelli M., Orsini F., Ferrero C., Percolating Hierarchical Defect Structures Drive Phase Transformation in Ce_1−x_GdxO_2−x/2_: A Total Scattering Study. *IUCrJ* **2015**, *2*, 511.

[5] Zhao C., Prosandeev S., Bellaiche L., Li F., Zhang S., Li S., Jones J. L., Bridging the Gap between the Short-Range to Long-Range Structural Descriptions of the Lead Magnesium Niobate Relaxor. *Acta Materialia* **2023**, *258*, 119171.

[6] Catalan G., Scott J. F., Physics and Applications of Bismuth Ferrite. *Advanced Materials* **2009**, *21*, 2463.

[7] Yoneda Y., Yoshii K., Kohara S., Kitagawa S., Mori S., Local Structure of BiFeO_3_–BaTiO_3_ Mixture. *Jpn. J. Appl. Phys.* **2008**, *47*, 7590.

[8] Otonicar M., Dragomir M., Rojac T., Dynamics of Domain Walls in Ferroelectrics and Relaxors. *Journal of the American Ceramic Society* **2022**, *105*, 6479.

[9] Damjanovic D., Stress and Frequency Dependence of the Direct Piezoelectric Effect in Ferroelectric Ceramics. *Journal of Applied Physics* **1997**, *82*, 1788.

[10] Hall D. A., Review Nonlinearity in Piezoelectric Ceramics. *Journal of Materials Science* **2001**, *36*, 4575.

[11] Bassiri-Gharb N., Fujii I., Hong E., Trolier-McKinstry S., Taylor D. V., Damjanovic D., Domain Wall Contributions to the Properties of Piezoelectric Thin Films. *J Electroceram* **2007**, *19*, 49.

[12] Bintachitt P., Jesse S., Damjanovic D., Han Y., Reaney I. M., Trolier-McKinstry S., Kalinin S. V., Collective Dynamics Underpins Rayleigh Behavior in Disordered Polycrystalline Ferroelectrics. *Proceedings of the National Academy of Sciences* **2010**, *107*, 7219.

[13] Robert G., Damjanovic D., Setter N., Turik A. V., Preisach Modeling of Piezoelectric Nonlinearity in Ferroelectric Ceramics. *Journal of Applied Physics* **2001**, *89*, 5067.

[14] Hall D. A., Stevenson P. J., High Field Dielectric Behaviour of Ferroelectric Ceramics. *Ferroelectrics* **1999**, *228*, 139.

[15] Davis M., Damjanovic D., Setter N., Direct Piezoelectric Effect in Relaxor-Ferroelectric Single Crystals. *Journal of Applied Physics* **2004**, *95*, 5679.

[16] Damjanovic D., Hysteresis in Piezoelectric and Ferroelectric Materials., in *The Science of Hysteresis*, Elsevier, **2006**, pp. 337–465.

[17] Morozov M. I., Damjanovic D., Hardening-Softening Transition in Fe-Doped Pb(Zr,Ti)O_3_ Ceramics and Evolution of the Third Harmonic of the Polarization Response. *Journal of Applied Physics* **2008**, *104*, 034107.

[18] Shvartsman V. V., Kholkin A. L., Domain Structure of 0.8Pb(Mg_1/3_Nb_2/3_)O_3_-0.2PbTiO_3_ Studied by Piezoresponse Force Microscopy. *Phys. Rev. B* **2004**, *69*, 014102.

[19] Shvartsman V. V., Kholkin A. L., Evolution of Nanodomains in 0.9PbMg_1/3_Nb_2/3_O_3_-0.1PbTiO_3_ Single Crystals. *Journal of Applied Physics* **2007**, *101*, 064108.
